# Mucosal ribosomal stress-induced PRDM1 promotes chemoresistance via stemness regulation

**DOI:** 10.1038/s42003-021-02078-1

**Published:** 2021-05-10

**Authors:** Juil Kim, Yuseok Moon

**Affiliations:** 1 Laboratory of Mucosal Exposome and Biomodulation, Department of Integrative Biomedical Sciences and Biomedical Research Institute, Pusan National University, Yangsan, Korea; 2 Graduate Program of Genomic Data Sciences, Pusan National University, Yangsan, Korea

**Keywords:** Cancer microenvironment, Pharmacodynamics

## Abstract

The majorities of colorectal cancer (CRC) cases are sporadic in origin and a large proportion of etiologies are associated with environmental stress responses. In response to external and internal stress, the ribosome stands sentinel and stress-driven ribosomal dysfunction triggers the cellular decision pathways via transcriptional reprogramming. In the present study, PR domain zinc finger protein (PRDM) 1, a master transcriptional regulator, was found to be closely associated with ribosomal actions in patients with CRC and the murine models. Stress-driven ribosomal dysfunction enhanced PRDM1 levels in intestinal cancer cells, which contributed to their survival and enhanced cancer cell stemness against cancer treatment. Mechanistically, PRDM1 facilitated clustering modulation of insulin-like growth factor (IGF) receptor-associated genes, which supported cancer cell growth and stemness-linked features. Ribosomal dysfunction-responsive PRDM1 facilitated signaling remodeling for the survival of tumor progenitors, providing compelling evidence for the progression of sporadic CRC.

## Abstract (web summary)

Kim and Moon find that the transcription factor PRDM1 is induced by ribosomal dysfunction in colorectal cancer (CRC) cells and associated with chemoresistance through a pathway involving IGF signaling. The authors further suggest PRDM1 promotes cancer cell stemness and survival, altogether providing insights into environmental stress-mediated signaling in CRC.

## Introduction

PR domain zinc finger protein (PRDM) 1, also known as beta-interferon gene positive-regulatory domain 1 binding factor (PRDI-BF1) or B lymphocyte-induced maturation protein (BLIMP-1), is a transcription factor that regulates a variety of genes to induce the differentiation of multiple effector cells^1,2^. PRDM1 was initially identified as a viral infection-induced repressor of transcription of interferon (IFN) β1^3^, which is mediated by recruiting the histone methyltransferase G9a, histone deacetylase^4,5^, or a co-repressor complex of Groucho family proteins^6^ to the promoter of the IFN-β gene. Moreover, PRDM1 inhibits IFN regulatory factor-dependent activation of the IFN-beta promoter by binding to the same motifs in IFN-β promoter^7^. Functionally, PRDM1 plays an essential role in the terminal differentiation of B cells into Ig-secreting plasma cells through direct transcriptional silencing of several key transcription activators critical for mature B cell identity and cellular proliferation^8^. The roles of PRDM family members, including PRDM1 have been implicated in human diseases, especially in hematological malignances^9,10^. For example, the *PRDM1* gene has been investigated as a tumor suppressor gene since it is located in chromosome 6q21–q22, which is frequently deleted from B cell lymphomas, including diffuse large B cell lymphoma^11^. However, it has been highly controversial for the involvement of PRDM1 in regulation of solid tumors, including colorectal cancer (CRC). For instance, while forced expression of PRDM1 inhibits clonogenic survival of primary colon tumor organoids in vitro^12^, it promotes human CRC cell growth through repression of p53 transcription^13^. Since the levels of PRDM1 in the normal gastrointestinal tract are too low to detect, it is needed to address the levels and actions of PRDM1 in the pathologic states of gut.

Mounting evidence support the concept that cancer is generally a polygenic multifactorial disease, which makes the environment an important modifier in the risk of CRC^14^. CRC has been widely considered as an environmental disease with a large proportion of cases from environmental factors, including dietary components, xenobiotics, and gut microbiota^15,16^. Upon exposure to internal or external stress, cells make decisions between death and survival. In particular, the ribosome stands sentinel to stress and stress-driven ribosomal dysfunction (ribosomal insult) triggers eukaryotic translation initiation factor 2 subunit α (eIF2α)-mediated global translational inhibition via protein kinase R (PKR), which is one of the primary integrated stress responses (ISR)^17–20^. Dysregulated translational control and somatic mutations in ribosomal proteins in cancer cells suggest the relevance of ribosomal dysfunction in tumorigenesis and cancer progression^21^. Mechanistically, in spite of ribosomal RNA injuries and subsequent eIF2α-mediated translational inhibition^17,19^, the ribosomal insult may result in the expression of genes important for cellular homeostasis and pathogenic processes for inflammation and chronic injuries^22–27^. Therefore, the ribosome plays crucial roles in sensing the stress from inner and external environment and its dysfunction triggers signals for cellular decisions depending on the degree of pathologic progress. However, little is known of stress-driven ribosomal dysfunction in the tumorigenesis and cancer cell responses to the oncogenic or anticancer factors. In the present study, PRDM1 expression was found to be closely associated with ribosomal actions in CRC patients. Based on this association, PRDM1-mediated modulation was evaluated as a platform signaling of cancer cellular responses under the ribosomal dysfunction. We assessed the model of ribosomal inactivation in CRC cells, which would provide novel insights into stress-gene crosstalk in the intestinal tumorigenesis and molecular evidence for interventions.

## Results

### PRDM1 expression is positively associated with intestinal tumorigenesis

**Fig. 1:**
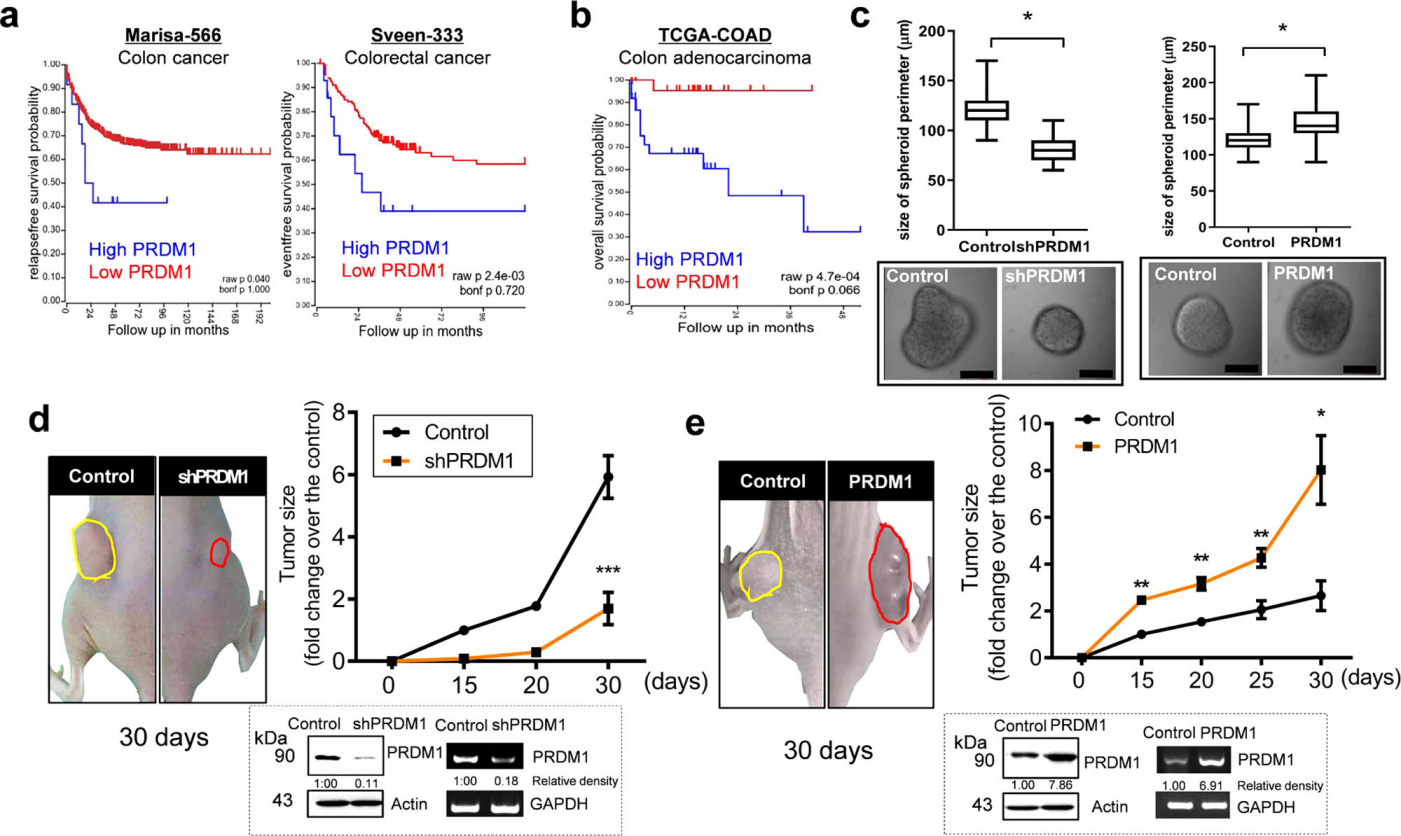
PRDM1 expression and roles in CRC. **a** Kaplan–Meier plot of survival analysis based on tissue *PRDM1* transcript levels in the patients with the colon cancer (left, *Marisa*’s, GEO ID: gse39582, *n* = 557; high *PRDM1* > 396.4, *n* = 12; low *PRDM1* < 396.4, *n* = 545) and colorectal cancer (right, *Sveen*’s, GEO ID: gse24551, *n* = 320; high *PRDM1* > 1073.1, *n* = 28; low *PRDM1* < 1073.1, *n* = 292). **b** Kaplan–Meier plot of survival analysis based on *PRDM1* expression level in patients with colon adenocarcinoma (TCGA-COAD, *n* = 155; high *PRDM1* > 1.755, *n* = 60; low *PRDM1* < 1.755, *n* = 95). **c** Control (the negative control shRNA or the empty vector pMX-IRES-GFP)-, shPRDM1-expressing HCT-8 (left) or PRDM1-overexpressing HCT-8 (right) (2 × 10^5^) were seeded in the ultralow attachment 6-well plates and cultured for 6 days, after which 200 spheroids per group were randomly selected and their average diameters measured. Results are shown as box-and-whisker plots (min to max) and asterisks (*) indicate significant differences from the control (*n* = 147, *P < 0.05 using two-tailed unpaired Student’s *t* test). The boxed images show representative spheroids. The microscopy analysis was performed at original magnification ×400; scale bar(s), 50 μm. **d**, **e** Control plasmid (the negative control shRNA (**d**) or pMX-IRES-GFP (**e**))-, shPRDM1 (**d**)-, or PRDM1 (**e**)-expressing HCT-8 (2 × 10^6^) were subcutaneously injected into BALB/c nude mice (left). Tumor size in mice was measured at each time after injection (right). Results are shown as mean values ± SD and asterisks represent a significant difference relative to the control group at each time point (*n* = 3, *P < 0.05, **P < 0.01, ***P < 0.001). Figures in the box indicate PRDM1 protein and mRNA expression in injected cancer cells using western blot analysis or RT-PCR.

Although PRDM1 is a well-known transcriptional regulator of the blood cancers, its contribution to the solid tumors are little established and controversial. Kaplan–Meier survival analysis using the clinical genomic datasets demonstrated that high levels of *PRDM1* levels are significantly associated with bad prognosis in the CRC patients (Fig. 1a). Since the collected cancer tissue samples can be heterogeneous, the colon adenocarcinoma-linked survival was particularly verified. Of note, patients with low expression of *PRDM1* in the colon adenocarcinoma displayed highly improved survival compared to the high expression group (Fig. 1b). Based on the histological category of the cancers, other carcinomas, sarcomas, and blood cancers, including lymphomas and myelomas were also assessed for *PRDM1*-linked survival of patients. Similar to the present results in colon adenocarcinoma, high level of *PRDM1* expression was also positively associated with poor prognosis in patients with carcinomas in the ovary, cervix, bladder, and esophagus (Supplementary Fig. 1A). In contrast, PRDM1 has been known as the tumor suppressor in the blood cancers, which was consistent in the present analyses on the survival of patients with lymphomas and myelomas (Supplementary Fig. 1B).

**Fig. 2:**
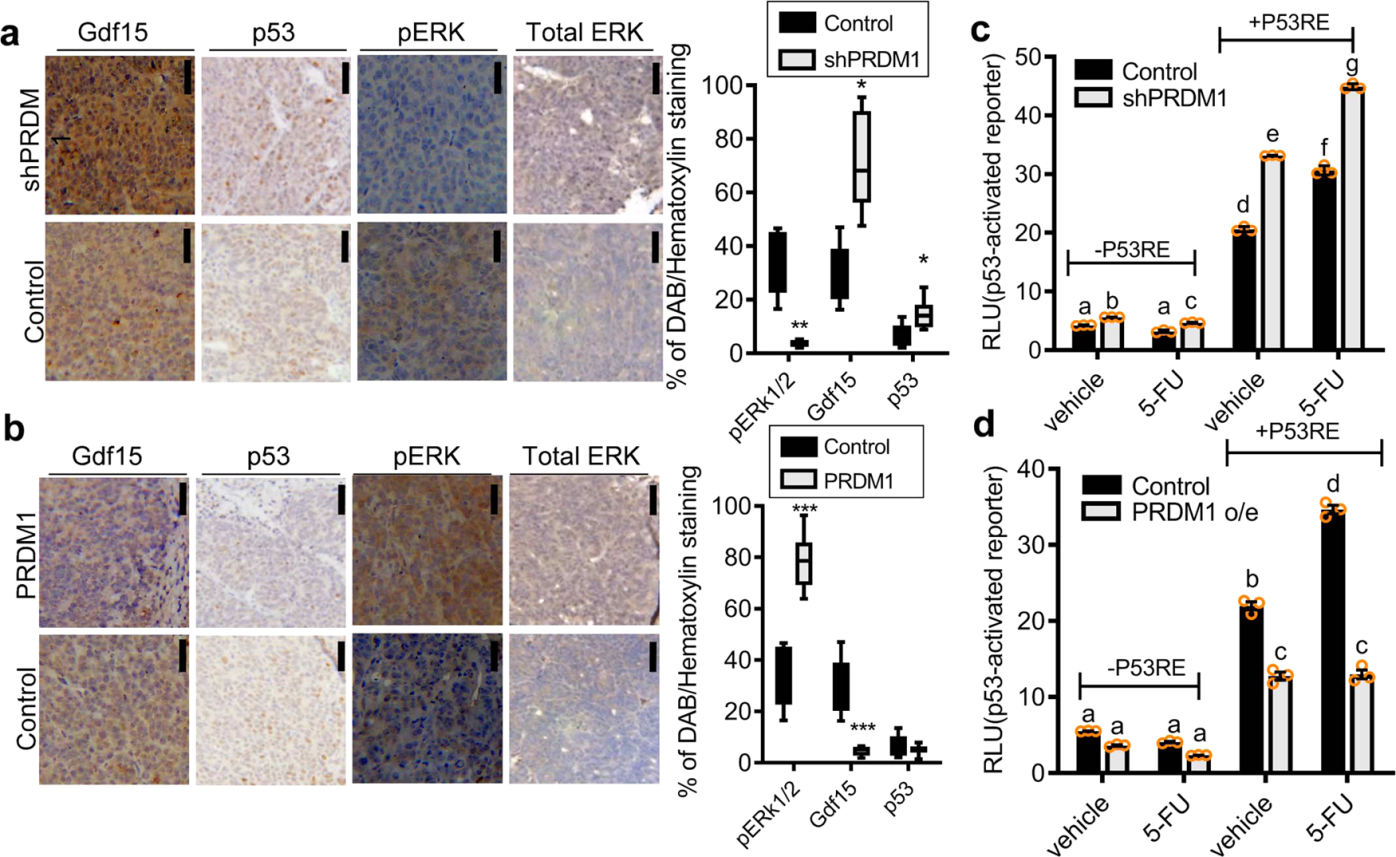
Tissue and transcriptional regulation of PRDM1-regulated signaling. **a**, **b** Control plasmid (the negative control shRNA (**a**) or pMX-IRES-GFP (**b**))-, shPRDM1 (**a**)-, or PRDM1 (**b**)-expressing HCT-8 (2 × 10^6^) were subcutaneously injected into BALB/c nude mice (left). Immunohistochemical (IHC) staining (×400; scale bar(s), 100 μm) of pERK1/2, Gdf15 and p53 and quantification of each gene in the tumor mass (right graphs). Results are shown as box-and-whisker plots (min to max) and asterisks represent a significant difference relative to the control group in each gene (*n* = 4–9, *P < 0.05, **P < 0.01, ***P < 0.001). **c**, **d** The intestinal cancer cells (the control plasmid-, shPRDM1 (**c**)-, PRDM1 overexpression (o/e) plasmid (**d**)-transfected HCT-8) were introduced with luciferase reporter plasmid with or without p53-responsive element (P53RE). Cells were treated with DMSO or 100 μM of 5-FU for 48 h and cellular luciferase activity was measured. Results are shown as mean values ± SD and different letters (a–g) over each bar represent significant differences between groups (*n* = 3, *P* < 0.05).

To recapitulate the evidence of PRDM1 actions in human CRC tissues, the growth of human intestinal cancer cells stably transfected with shRNA- or PRDM1-expression vector (denoted as shPRDM1 or PRDM1, respectively) was tested by measuring the cancer spheroid formation in vitro. PRDM1 overexpression increased the size of CRC spheroids, whereas genetic knockdown of PRDM1 interfered with the growth of the spheroids (Fig.1c). These tumor growth-promoting actions of PRDM1 were also verified in an ectopic xenograft model (Fig. 1d, e). While genetic knockdown of PRDM1 expression decreased tumor growth (Fig. 1d), overexpression of PRDM1 significantly enhanced the tumor size in the xenograft animals (Fig. 1e). Moreover, PRDM1-mediated alteration of cancer cell signal was assessed in tissues of the xenograft tumor model. Notably, suppression of PRDM1 attenuated mitogenic extracellular signal-regulated kinase (ERK) 1 and 2 phosphorylation in the tumor mass, whereas levels of pro-apoptotic p53 and growth/differentiation factor 15 (Gdf15), a representative target of p53 signal, were elevated (Fig. 2a). Conversely, ectopic PRDM1 overexpression reduced expression of pro-apoptotic molecules, including Gdf15 and p53, with subsequent enhancement of active ERK1/2 (Fig. 2b). The inverse correlation between PRDM1 and p53 signaling was mechanistically verified using p53-responsive luciferase reporter system in the intestinal cancer cells. While genetic knockdown of PRDM1 elevated p53-responsive reporter activity, PRDM1 overexpression downregulated the reporter activity (Fig. 2c and d). Furthermore, p53-mediated reporter levels were assessed after treatment with 5-fluorouracil (5-FU) as a conventional chemotherapeutic agent, one of the first line anticancer drug against CRC. The chemotherapeutic agent increased p53-mediated reporter expression, which was also counteracted by PRDM1 (Fig. 2c and d). All of these evidence indicate that PRDM1 is a negative regulator of p53-responsive elements in both intact and 5-FU-treated cancer cells. Taken together, high tissue expression of PRDM1 indicates the bad prognosis of patients with CRC, particularly, the colon adenocarcinoma. Experimentally, adenocarcinoma-derived PRDM1 was positively involved in the tumor mass increase. The clinical survival analysis and xenograft models support the positive regulation of tumor growth by PRDM1 in the CRC.

### Ribosomal dysfunction is closely associated with PRDM1 in CRC cells

**Fig. 3:**
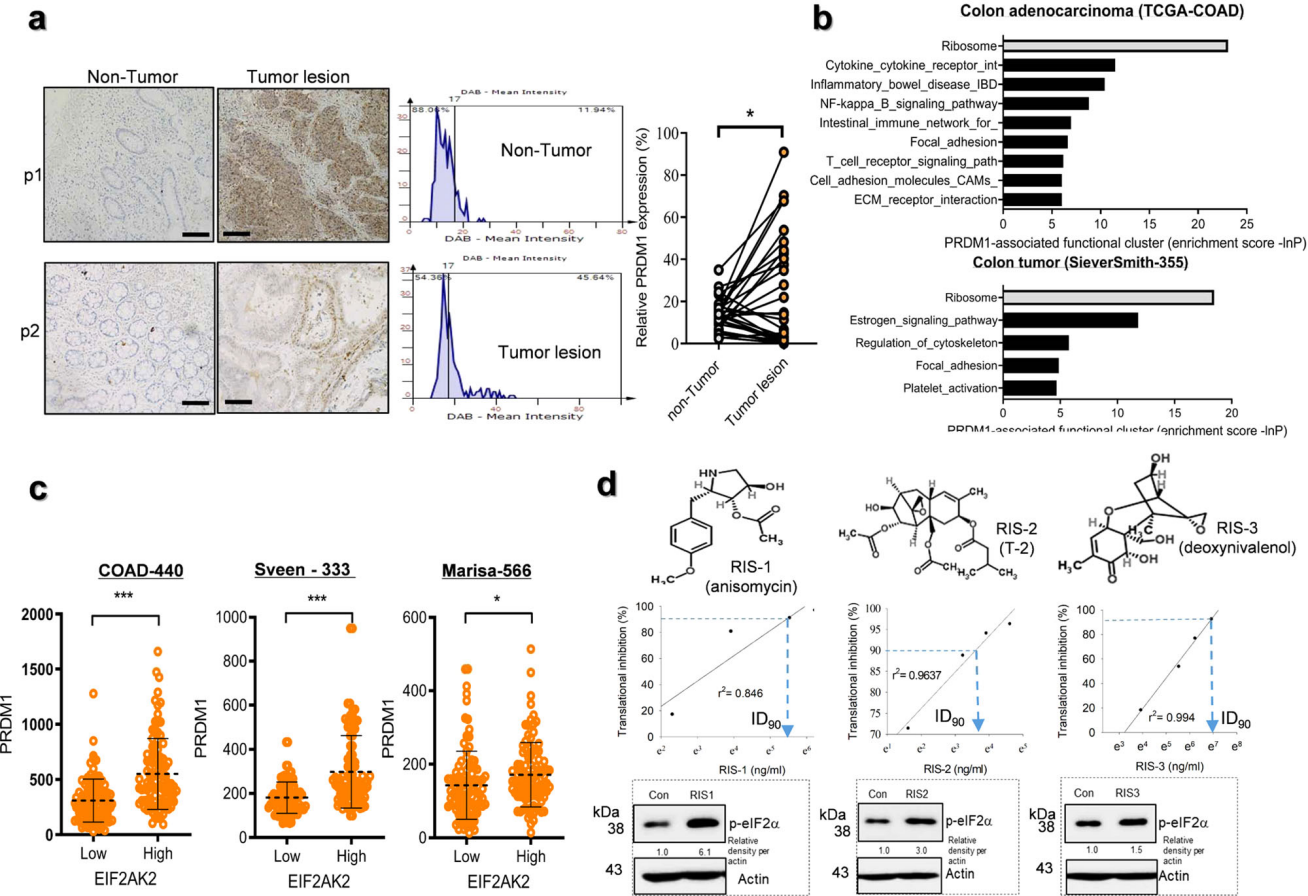
PRDM1 expression and ribosomal stress in CRC. **a** PRDM1 immunohistochemistry (IHC) of normally appearing parts (left) and tumor lesions (right) from each patient with quantification of PRDM1 staining from IHC analysis using Histo-quest software 4.0 (the mid histograms). All patients’ quantitation was statistically analyzed (the right graph). Asterisks indicate a significant difference compared to each non-tumor lesion based on the paired *t-*test (*n* = 30, *P* < 0.05). The microscopy analysis was performed at original magnification ×200; scale bar(s), 50 μm. **b** KEGG-based functional annotations of PRDM1-related genes in CRC patients (TCGA COAD (*n* = 286, upper graph), and SieverSmith (gse14333 and gse17538, *n* = 355, lower graph)). PRDM1-associated genes in CRC patients were selected using the correlation analysis (R cutoff = absolute 0.5) and HugoOnce algorithm. Selected genes were clustered based on KEGG pathway analysis (*P* < 0.05) and the enrichment score was described as values of −ln(P). **c** Expression of *PRDM1* was assessed in patients with CRC (TCGA-COAD, *n* = 440; Sveen’s, gse24551, *n* = 333; Marisa’s, gse39582, *n* = 566). Based on *EIF2AK2* levels, we chose the 100 highest and 100 lowest level samples, which were further compared for levels of *PRDM1*. Results are shown as mean values ± SD and asterisks (∗) indicate significant differences from the low expression group (*P < 0.05, ***P < 0.001). **d** Chemical structures of RIS-producing agents (upper panels). HCT-8 cells were treated with serial concentrations of RIS for 24 h, and changes in total protein synthesis per a cell from 0 to 24 h were measured (middle graphs). The additional translational arrest was verified by detecting p-eIF2α levels in the ribosome-inactivated cells (lower panels for western blot analysis).

**Fig. 4:**
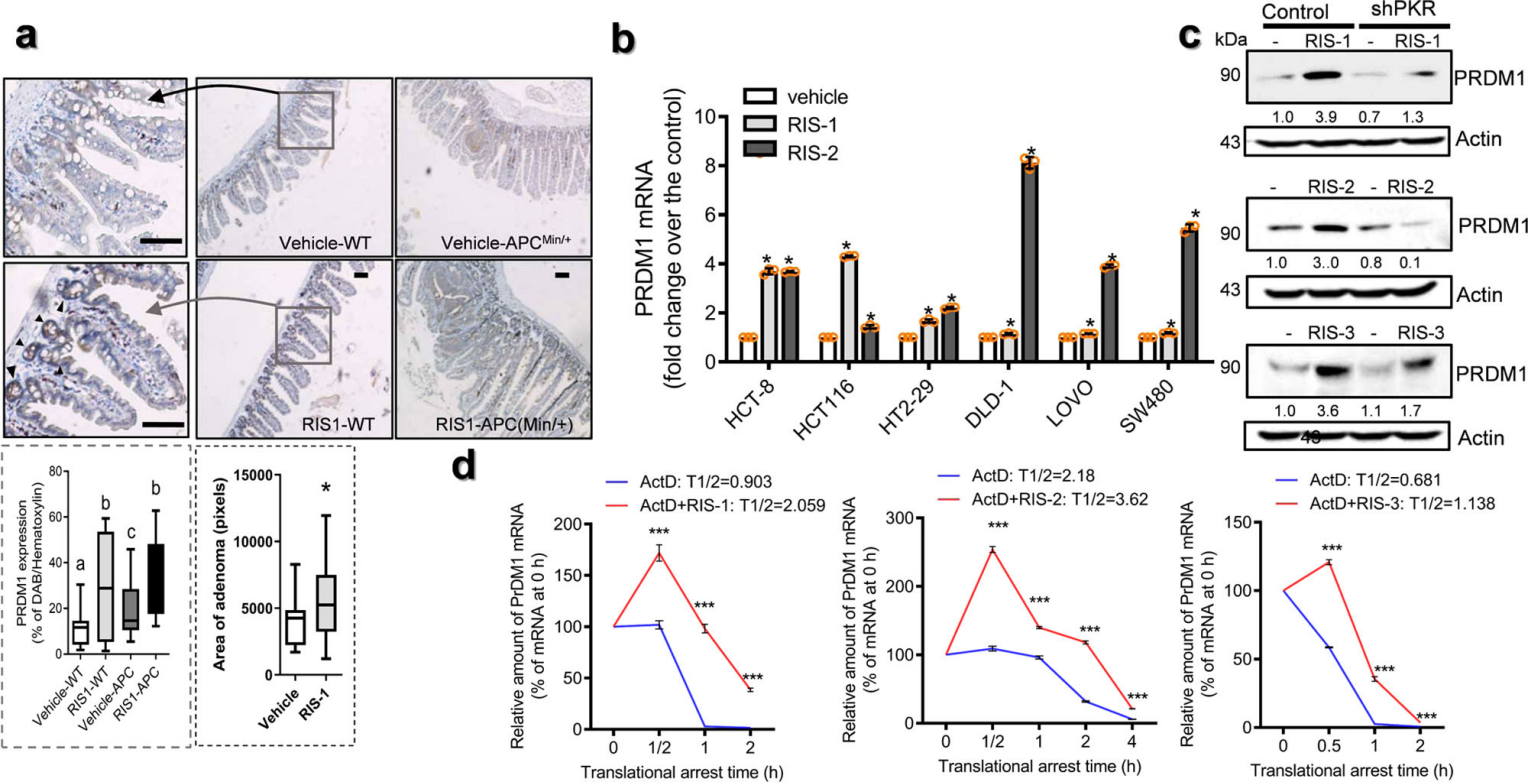
PRDM1 expression in ribosome-inactivated CRC cells. **a** Wild type or APC^Min/+^ mice (*n* = 3–5) were treated with 25 mg/kg RIS-1 for 72 h, after which the PRDM1 levels in the small intestine were measured using immunohistochemistry with hematoxylin staining (magnification ×100; scale bar(s), 50 μm). The arrows indicate higher levels of PRDM1 expression in the crypt than in the villi. Quantification of PRDM1 staining from IHC analysis (the lower graph) and relative area of adenoma in the gut. Results are shown as a plot with Tukey whiskers and different letters (a–c) over each box represent significant differences between groups (the lower left graphs, *n* = 12–20, *P* < 0.05). Asterisks represent a significant difference compared to each vehicle group (the lower right graph, *n* = 16–39, *P* < 0.05). **b** *PRDM1* mRNA expression was measured in the intestinal cancer cells treated with ID_90_ of RIS-1 or RIS-2 for 2 h. Results are shown as mean values ± SD and asterisks represent a significant difference compared to each vehicle group (*n* = 3, *P* < 0.05). **c** HCT-8 cells were transfected with control (the negative control shRNA) or shPKR plasmid. PRDM1 protein was detected in the HCT-8 cells treated with RIS-1 for 6 h, RIS-2 for 24 h or RIS-3 for 6 h (different times indicate points for each maximal expression of PRDM1). Total cell lysates were subjected to western blot analysis. **d** HCT-8 cells were treated with ID_90_ of RIS for 8 h (RIS-1 or RIS-3) or for 2 h (RIS-2) and then added 5 μM actinomycin D for the indicated time to arrest cellular transcription. Each mRNA level was measured using RT real-time PCR. Results are shown as mean values ± SD and asterisks (∗) indicate significant differences from the group without RIS treatment at each time point (*n* = 3, ***P < 0.001).

Tissue expression analysis of PRDM1 in biopsies of 30 CRC patients demonstrated that the basal levels of PRDM1 expression in normally-appearing parts of the gut were too low to detect (Fig. 3a and Supplementary Fig. 2). The paired *t*-test revealed significant increase in PRDM1 expression in tumor lesions, compared to the levels in the non-tumor regions (Fig. 3a, *p* < 0.05, the right graph). Next, we performed functional estimation of PRDM1-linked events. *PRDM1*-involved genes were selected from tissue transcriptome of CRC patients using clinical datasets and their functional evaluation with KEGG pathway analysis demonstrated the ribosome-associated events are closely linked to *PRDM1* in CRC, particularly the colon adenocarcinoma (Fig. 3b). The ribosome is a crucial sentinel organelle in response to internal or external environment and stress-driven ribosomal dysfunction triggers ISR via PKR in pathogenic states. Clinical transcriptomic analysis indicated that CRC patients with high levels of EIF2AK2 (PKR) as the key modulator of ribosomal stress responses display increased levels of *PRDM1* (Fig. 3c). Therefore, it was hypothesized that PRDM1 is involved in stress responses of the intestinal tumor cells. Stress-driven ribosomal dysfunction was induced by treatment with specific ribosome-inactivating stress (RIS) agents such as anisomycin (RIS-1), T-2 toxin (RIS-2), and deoxynivalenol (RIS-3) which specifically induce eIF2α-mediated global translational inhibition via PKR. First, they were confirmed for in vitro protein synthesis inhibitory actions via eIF2α-linked signaling in CRC cells (Fig. 3d). To verify the stress responses in CRC, stress-driven ribosomal dysfunction was further applied in the genetic model of familial adenomatous polyposis using Min (multiple intestinal neoplasia) mice which carry a truncation mutation of the *Adenomatous polyposis coli (APC)* gene (APC^Min/+^). Consistent with elevation of PRDM1 expression in tumor tissues of the CRC patients, the protein levels were higher in the gut of APC^Min/+^ mice than in that of the wild type normal mice (Fig. 4a). Of note, ribosomal dysfunction significantly increased PRDM1 expression in intestinal epithelia and adenomas, leading to particularly high levels in the crypt parts (Fig. 4a). In addition to the tissue levels, the in vitro ribosomal dysfunction enhanced *PRDM1* mRNA levels in different types of intestinal cancer cells (Fig. 4b). Moreover, ribosomal dysfunction-induced PRDM1 was dependent on PKR as a central eIF2α kinase of integrated stress responses in the intestinal cancer cells (Fig. 4c). Despite of the global translational inhibition, ribosomal dysfunction increased PRDM1 protein levels via enhanced stability of *PRDM1* mRNA (Fig. 4d), potently increasing total level of *PRDM1* transcript and overcoming the action of reversible translation arrest with time.

### PRDM1 is an essential survival factor of chemoresistance via up-regulation of the IGF-linked signaling pathway in the colon cancer cells

**Fig. 5:**
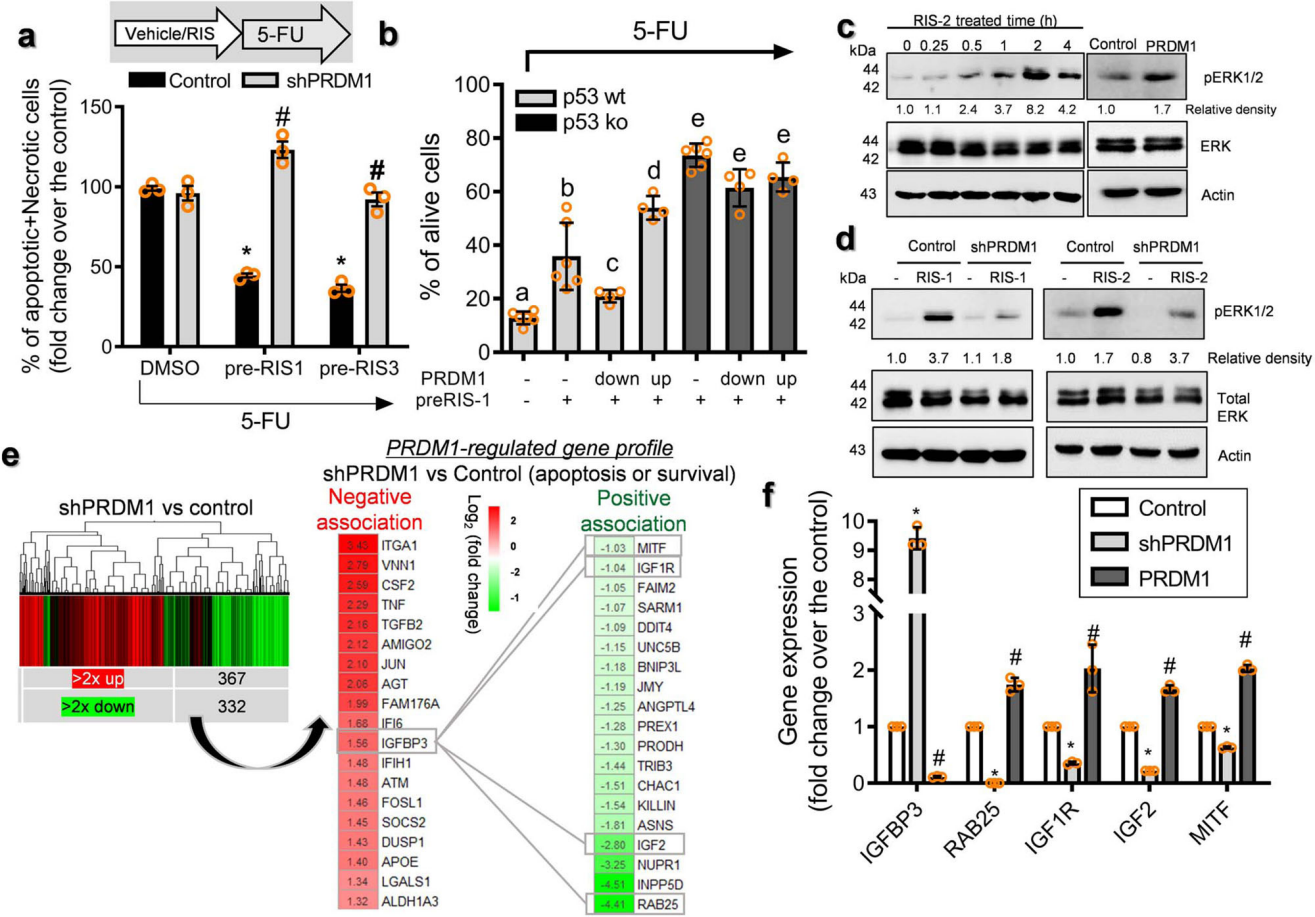
Roles of PRDM1 in chemoresistance and related signals. **a** The intestinal cancer cell lines (the negative control plasmid- or shPRDM1-expressing HCT-8) pre-exposed to ID_80_ of RIS-1 or RIS-3 for 24 h were treated with 375 μM 5-FU for 48 h. Results are shown as mean values ± SD. Asterisks represent a significant difference compared to each vehicle (DMSO) treatment group and the symbols (#) indicate a significant difference compared to each control cells (*n* = 3, *P* < 0.001). **b** The intestinal cancer cells (the control plasmid-, shPRDM1 or PRDM1-expressing HCT-116) pre-exposed to ID_80_ of RIS-1 or RIS-3 for 24 h were treated with 375 μM 5-FU for 48 h. Alive cells were counted with a hemocytometer and different letters (a–d) over each bar represent significant differences between groups (*n* = 4–6, *P* < 0.05). **c** HCT-8 cells were treated with ID_90_ of RIS-2 for the indicated times and total cell lysates were subjected to western blot analysis. **d** The intestinal cancer cell lines (the negative control plasmid- or shPRDM1-expressing HCT-8) were treated with ID_90_ of RIS-1 for 30 min (the left panels) or ID_90_ of RIS-2 for 2 h (the right panel). Total cell lysates were subjected to western blot analysis. **e** Gene expression profile in cancer cell lines (the negative control vector- or shPRDM1-expressing HCT-8) using comparative cDNA microarray measurements (*n* = 3, *p* < 0.05, left). 2× up or 2× down related genes involved in apoptosis or survival response (*n* = 3, *P* < 0.05, right). **f** The relative mRNA expressions in cell lines (control plasmid (the negative control shRNA or the empty control pMX-IRES-GFP)-, shPRDM1-, or PRDM1-expressing HCT-8) were quantified using RT real-time PCR. Results are shown as mean values ± SD. Asterisks or sharps represent a significant difference (*n* = 3, *P* < 0.05) relative to each control group.

In addition to the tumor growth-promoting actions of PRDM1 (Fig. 1), we further assessed its contribution to cancer cell survival in a harsh condition, such as anticancer stress. PRDM1-mediated action was thus analyzed following treatment with 5-FU. Specifically, ribosome-inactivated CRC cells were treated with 5-FU. When compared with non-insulted cancer cells, ribosome-inactivated cells showed notable resistance to the cytotoxic actions of 5-FU (Fig. 5a and Supplementary Fig. 3a). Moreover, genetic knockdown of PRDM1 attenuated RIS-driven chemoresistance whereas PRDM1 overexpression improved cancer cell survival (Fig. 5b), indicating that cellular ribosomal stress mitigates conventional chemotherapeutic actions via PRDM1 protein. Furthermore, p53 was evaluated as a potent component involved in PRDM1-mediated cellular responses since PRDM1 counteracted p53-responsive transcriptional activity (Fig. 2c and d). Genetic ablation of p53 increased ribosomal dysfunction-driven chemoresistance even when PRDM1 expression was suppressed (Fig. 5b). All evidence indicate that PRDM1 counteracts p53-mediated cytotoxicity of chemotherapeutic 5-FU in the ribosome-inactivated cancer cells.

Given that PRDM1 is required for cancer cell survival and inhibits anticancer actions, PRDM1 can be expected to trigger cell growth factor-linked survival signals. Among these, ERK 1 and 2 have been shown to be representative growth factor-responsive mitogen-activated protein kinases (MAPK) that are involved in various growth factor-linked cellular processes and are activated by ribosomal inactivation as well^22,28–30^. Intestinal cancer cells showed enhanced phosphorylation of ERK1/2 in a time-dependent manner in response to chemical ribosomal inactivation in vitro (Fig. 5c). Moreover, PRDM1 overexpression also activated ERK1/2 signals in cancer cells. Ribosomal dysfunction-activated ERK1/2 was notably attenuated by PRDM1 depletion via its shRNA, indicating the positive regulation of ERK1/2-linked signaling pathways by PRDM1 in response to ribosomal dysfunction in cancer cells (Fig. 5d).

**Fig. 6:**
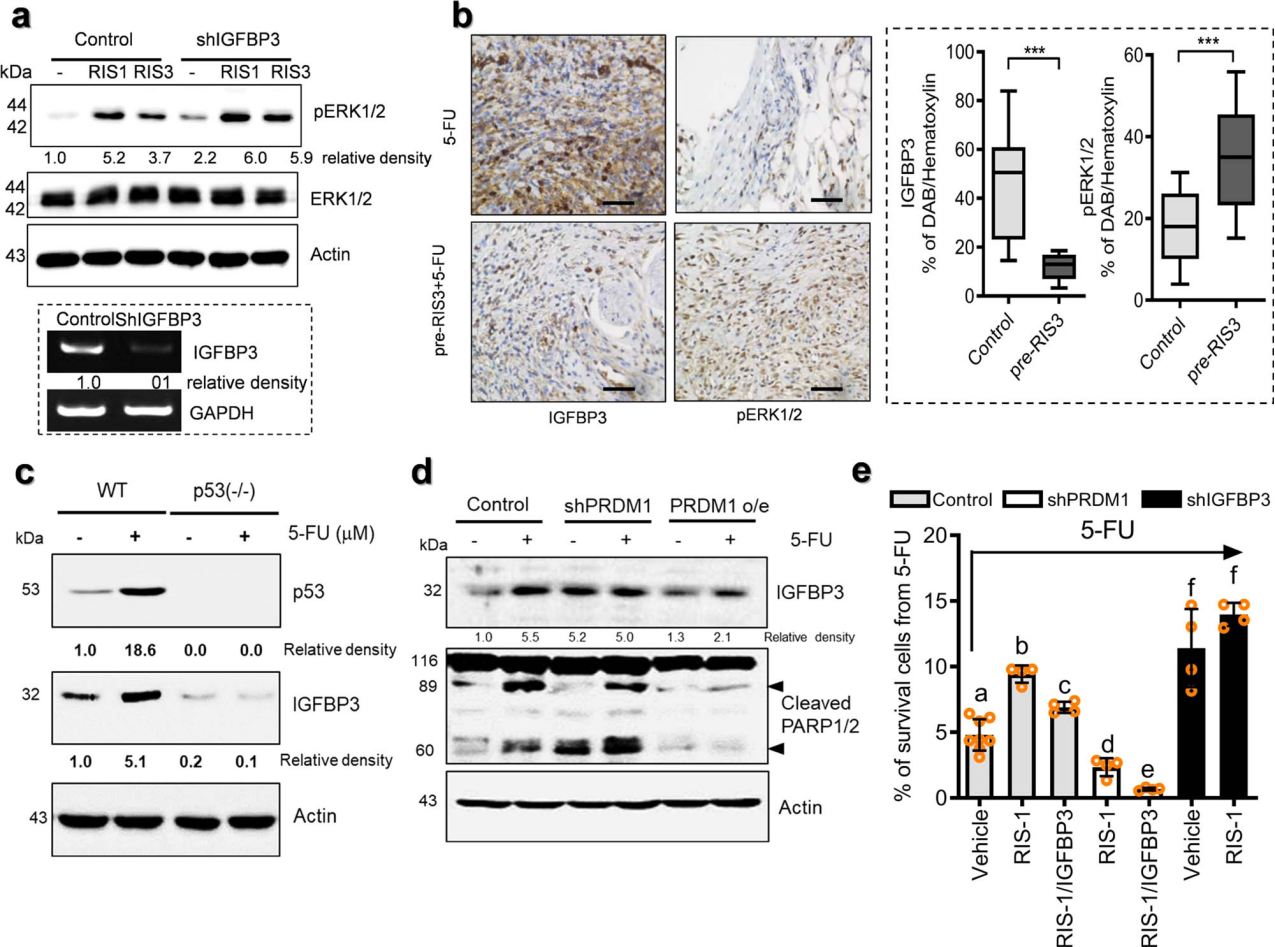
Roles of IGFBP3 in chemoresistance. **a** The intestinal cancer cell lines (the negative control plasmid- or shIGFBP3-expressing HCT-8) were treated with DMSO, ID_90_ of RIS-1 or ID_90_ of RIS-3. Total cell lysates were subjected to western blot analysis. Figures in the lower box indicate IGFBP3 mRNA expression in cell lines. **b** Mouse tissues from allograft CRC were analyzed using immunohistochemistry for IGFBP3, and pERK1/2 (left panels) with hematoxylin counterstaining (magnification ×400; scale bar(s), 100 μm). Quantification of DAB staining from IHC analysis (right graphs). Results are shown as a plot with Tukey whiskers and asterisks represent a significant difference compared to the control group (*n* = 11 or 14, ***P *<* 0.001). **c** p53 wildtype (+/+) and mutant (−/−) HCT-116 cells were treated with DMSO or 5-FU for 48 h. Total cell lysates were subjected to western blot analysis. **d** The intestinal cancer cells (the control vector-, shPRDM1- or PRDM1 overexpression plasmid-transfected HCT-8 cells) were treated with DMSO or 375 μM 5-FU for 48 h. Total cell lysates were subjected to western blot analysis. **e** The intestinal cancer cell lines (the control plasmid-, shPRDM1- or shIGFBP3-expressing HCT-8) pre-exposed to the vehicle, ID_80_ of RIS-1 or 50 ng/ml IGFBP3 for 24 h were treated with 375 μM 5-FU for 48 h and mRNA levels were measured using RT real-time PCR. Results are shown as mean values ± SD and different letters (a–f) over each bar represent significant differences between groups (*n* = 4–8, *P* < 0.05).

We next performed comparative genome-wide microarray analysis in PRDM1-suppressed HCT-8 intestinal cancer cells to investigate the molecular mechanism of PRDM1-mediated cell survival in association with ERK1/2 signals. We selected 699 candidates that were up- or downregulated by over 2-fold relative to the control group (Fig. 5e, left) and further addressed the functional signature of cell survival and apoptosis (Fig. 5e, right). Closer comparison of selected candidates notably identified clustering regulation of IGF signaling-related components. In particular, *Ras-related protein 25 (RAB25)*, *IGF2*, and *IGF1 receptor (IGF1R*) were suppressed in PRDM1-depleted HCT-8 cells whereas *insulin-like growth factor binding protein 3 (IGFBP3)* was elevated (Fig. 5e). We evaluated the alteration of gene expression of selected candidates by quantitative real-time RT-PCR. In a similar way to microarray analysis, the quantitative PCR demonstrated that IGFBP3 level was increased by over 9-fold in PRDM1-depleted cells while the levels of *RAB25, IGF1R, IGF2*, and *microphthalmia-associated transcription factor (MITF)* are positively associated with PRDM1 (Fig. 5f). In clinical and experimental evidence, low IGF bioactivity is associated with protection against tumor development and metastasis, suggesting that IGFs provide potent pro-tumorigenic signals^31^. In terms of PRDM1, the expression of IGFBP3 was inversely related to that of RAS-related signaling molecules, including RAB25, IGF1R, IGF2, and MITF. Since HCT-8 cells have a *KRAS* active mutation, noncancerous HEK293 cells with no *KRAS* mutation were also compared for the effects of PRDM1 expression on IGF-RAS-related signaling molecules (Supplementary Fig. 3b–c). In HEK293 cells, the expression of IGF-RAS-related signaling molecules was also positively associated with PRDM1 expression. Among the IGF signal-linked genes identified in the present study, PRDM1 downregulated expression of IGFBP3 and thus the effects of IGFBP3 suppression on the ERK1/2 signal as central downstream signaling kinases of IGF-RAS-linked pathways were assessed in ribosome-inactivated cancer cells. Notably, ERK1/2 activation in response to ribosomal dysfunction was enhanced by IGFBP3 depletion, indicating negative regulation of ERK1/2-linked signals by IGFBP3, an endogenous antagonist of IGF receptor (Fig. 6a). Furthermore, IGF-linked signals were assessed in the CRC tissues from tumor cell allograft mice treated with the chemotherapeutic agent. Consistent with the in vitro expression evidence, ribosomal dysfunction enhanced the survival-linked ERK1/2 signals, downregulating expression of the anticancer-related IGFBP3 in cancer cells under chemotherapeutic action (Fig. 6b). Based on these results, it was hypothesized that IGF receptor-linked signaling mediates ribosomal stress-induced tumor cell survival from anticancer action of 5-FU. In contrast with survival-linked ERK1/2 signaling, IGFBP3 was positively regulated by apoptosis-associated p53 in response to 5-FU treatment (Fig. 6c). Consistent with the genome-wide microarray data of human CRC cells (Fig. 5e), PRDM1 counteracted IGFBP3 expression and chemotherapeutic agent-induced apoptosis (Fig. 6d). In particular, IGFBP3 significantly downregulated ribosomal dysfunction-enhanced tumor cell survival (Fig. 6e). In contrast, IGFBP3-suppressed cells displayed enhanced survival in response to 5-FU. Taken together, the ribosomal dysfunction-triggered PRDM1-mediated survival of CRC cells via IGF-linked signaling pathways, which functionally counteracted anticancer action.

### PRDM1-IGF signals mediate CRC cell stemness and subsequent survival

**Fig. 7:**
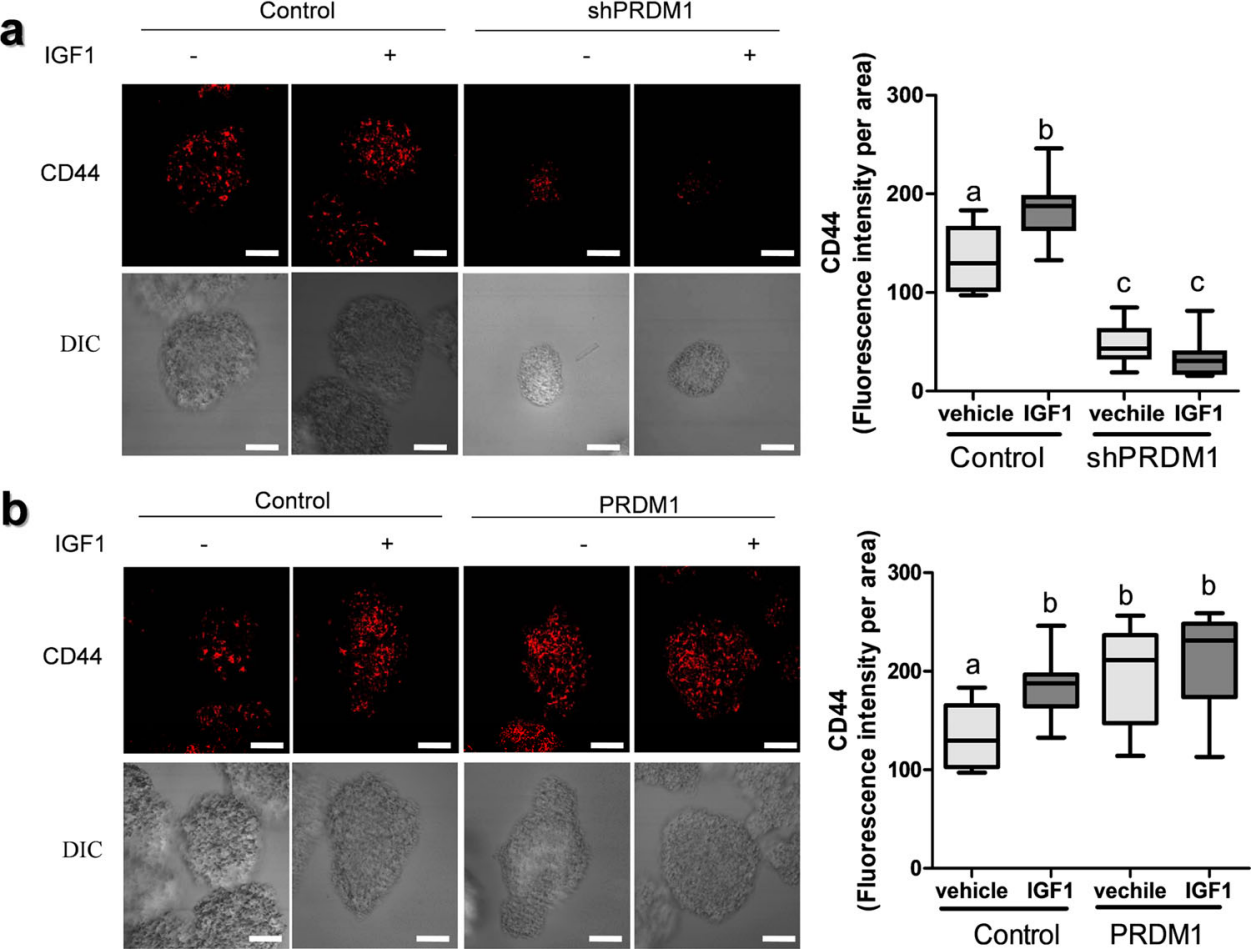
Effects of PRDM1 and IGF signaling on stemness biomarkers in human CRC cell spheroids. **a**, **b** Spheroids from human CRC cells (control (the negative control shRNA (**a**) or the empty vector pMX-IRES-GFP (**b**))-, shPRDM1-expressing HCT-8 (**a**) or PRDM1-overexpressing HCT-8 (**b**)) were treated with DMSO or 25 ng/ml IGF1 for 6 days, then immunostained against CD44 (red). The microscopy analysis was performed at original magnification ×400; scale bar(s), 50 μm. CD44 fluorescence was quantified using the Image J software (the right graphs). Results are shown as a plot with Tukey whiskers and different letters (a–c) over each box represent significant differences between groups (*n* = 10, *P* < 0.05).

**Fig. 8:**
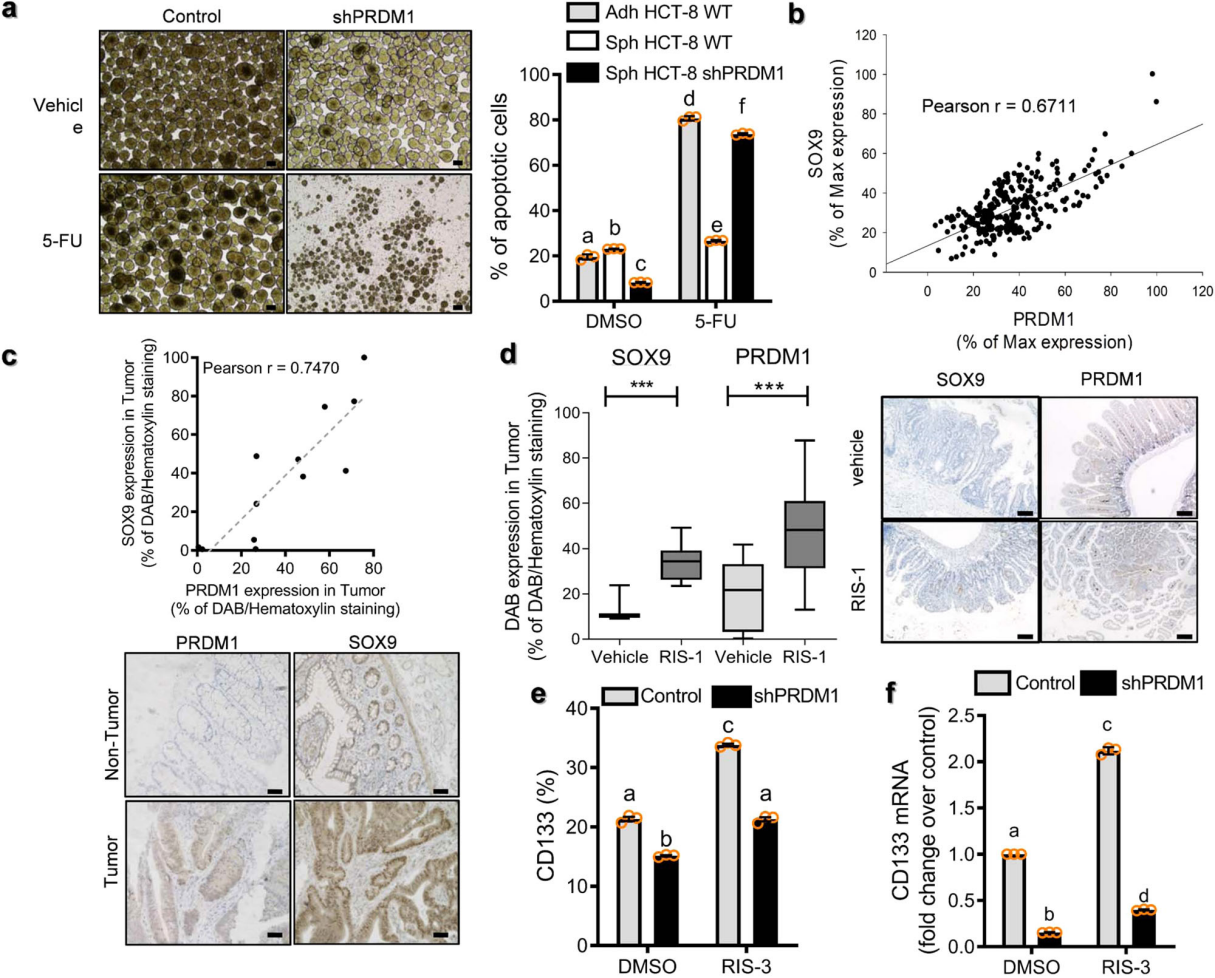
Correlation between PRDM1 and stemness biomarkers in human CRC tissues and APC^Min/+^ mice. **a** The intestinal cancer cell lines (the negative control plasmid- or shPRDM1-expressing HCT-8) were treated with DMSO or 375 μM 5-FU in the state of adherent cells (Adh) and spheroids (Sph). Cellular apoptosis was quantified (the right graph) and spheroids were visualized (the left). Results are shown as mean values ± SD and different letters (a–f) over each bar represent significant differences between groups (*n* = 3, *P* < 0.05). The microscopy analysis was performed at original magnification ×100; scale bar(s), 50 μm. **b** Correlations between SOX9 and PRDM1 mRNA expression in CRC patients (TCGA 286, ID: COAD, *n* = 286) were calculated using a two-tailed Pearson correlation test. **c** Histological sections of human CRC tissues were analyzed by immunohistochemistry against PRDM1 and SOX9. Correlations between SOX9 and PRDM1 protein expression in CRC patients (*n* = 12) were calculated using a two-tailed Pearson correlation test. The representative microscopic observation was demonstrated at the original magnification ×200; scale bar(s), 50 μm (lower panels). **d** APC^Min/+^ mice received the vehicle or 25 mg/kg RIS-1 via oral gavage and at 72 h later the small intestine was examined by immunohistochemistry. Results are shown as a plot with Tukey whiskers and asterisks represent a significant difference relative to the vehicle group (*n* = 3–15, ***P < 0.001). The representative microscopic observation was demonstrated at the original magnification ×100; scale bar(s), 50 μm (right panels). **e**, **f** Human CRC cell spheroids from the negative control plasmid- or shPRDM1-expressing HCT-8 (2 × 10^5^) were treated with ID_90_ of RIS-3 for 24 h to assess levels of each marker. CD133-positive populations of CD133 (**e**) and mRNA levels of *CD133* (**f**) were measured. Results are shown as mean values ± SD and different letters (a–c) over each bar represent significant differences between groups (*n* = 3, *P* < 0.05).

Since cancer stemness is the determinative factor of chemoresistance in various types of cancers including CRC^32,33^, PRDM1 was assessed for its effects on formation of cancer stem cells (CSCs). Moreover, expression of PRDM1 in ribosome-inactivated gut was notably high in the intestinal crypts (Fig. 4a) which are stem cell-rich regions. The growth of CSCs can be facilitated by three-dimensional culture of cancer cells such as spheroid culture since CSCs in spheroids are more able to overexpress “stemness” genes^34,35^. In the present study, CRC cell spheroid-derived stemness was measured using anti-CD44 antibody (Fig. 7a). In particular, CD44 levels were significantly elevated by IGF treatment, which was attenuated by knockdown of PRDM1. In contrast, overexpression of PRDM1 enhanced levels of CRC stemness marker CD44 (Fig. 7b). Since PRDM1 can induce components involved in IGFR-linked pathway, overexpression of PRDM1 displayed saturated levels of IGF-elevated cancer stemness (Fig. 7b). All of these evidence indicate that PRDM1-enhanced IGF signaling play key roles in displaying the feature of CRC cell stemness. In consistency with an assumption that the cancer stemness contributes to cell survival from anticancer actions, we demonstrated that CSC stem cell-rich spheroids were more resistant to 5-FU than conventionally adherent CRC cells (Fig. 8a). In contrast, PRDM1-depletion increased the chemosensitivity to 5-FU in cancer spheroids (Fig. 8a), indicating that CRC stemness was positively associated with PRDM1-mediated chemoresistance. The clinical implications of PRDM1 in cancer stemness were also observed in tumor tissues from CRC patients. Transcriptomic dataset analysis demonstrated that tumor-derived PRDM1 was positively associated with expression of Sox-9, a well-known modulator of cancer progenitor cells in CRC (Fig. 8b). Consistent with the evidence in the CRC transcriptomic dataset, *PRDM1*-high expression group displayed enhanced protein levels of *Sox-9* in CRC tissues with a positive correlation (Fig. 8c).

**Fig. 9:**
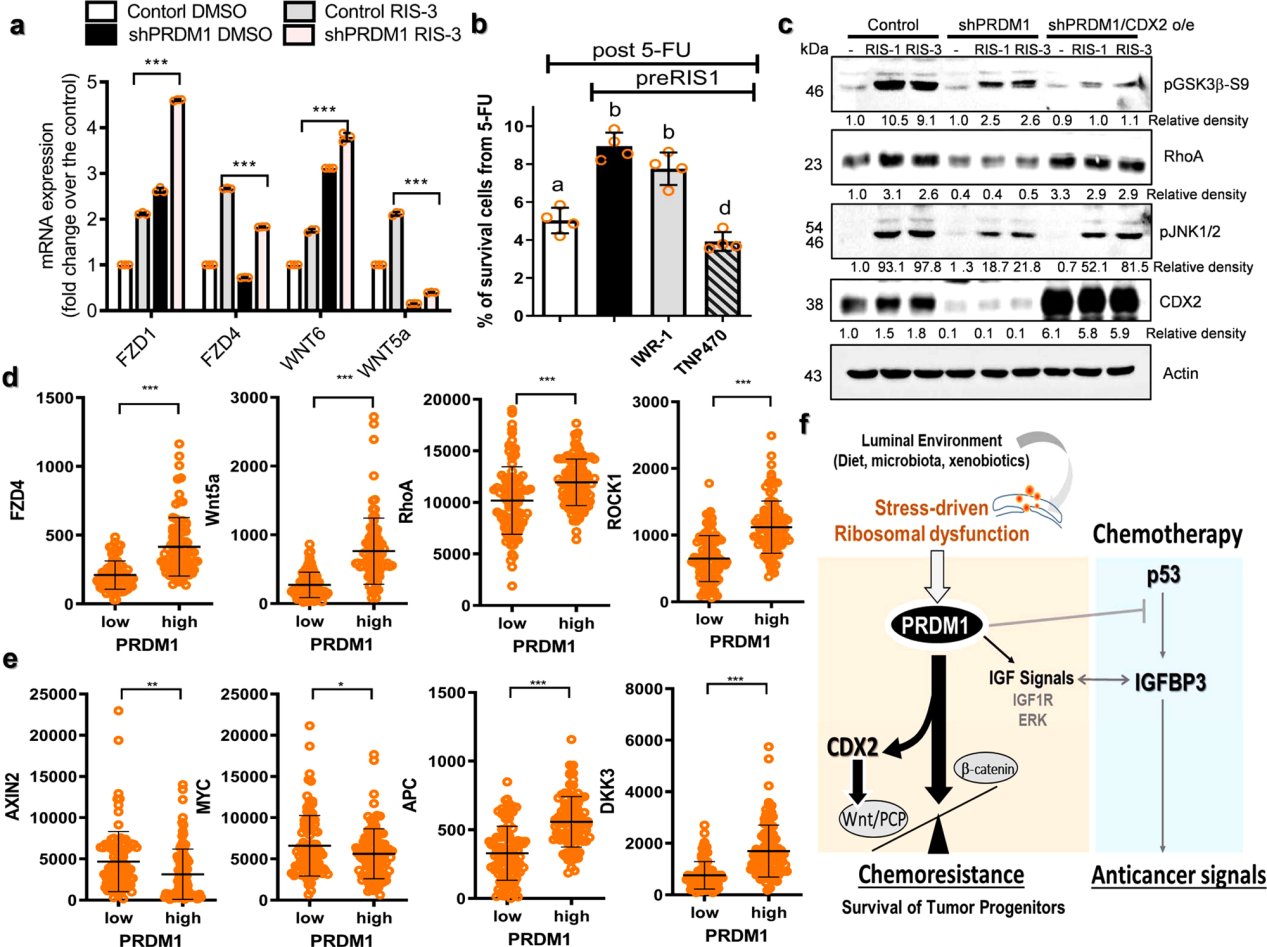
Stemness-linked Wnt signals in ribosome-inactivated CRC. **a** Human CRC cell spheroids from the control plasmid- or shPRDM1-expressing HCT-8 (2 × 10^5^) were treated with ID_90_ of RIS-3 for 24 h to assess levels of each marker. mRNA levels of Wnt-related genes were measured (*n* = 3, ***P < 0.001). **b** HCT-8 cells pre-exposed to ID_80_ of RIS-3 along with 30 μM IWR-1 or 10 μM TNP470 for 24 h were treated with 375 μM 5-FU for 48 h. Results are shown as mean values ± SD and different letters (a–c) over each bar represent significant differences between groups (*n* = 4, *P* < 0.05). **c** The intestinal cancer cells (the control vector-, shPRDM1- or shPRDM1 + CDX2 overexpression plasmid-transfected HCT-8) were treated with DMSO, ID_90_ of RIS-1 or RIS-3 for 30 min. Total cell lysates were subjected to western blot analysis. **d**, **e** Expressions of Wnt-linked genes were assessed in patients with CRC (TCGA-COAD, *n* = 440). Based on *PRDM1* levels, we chose the 100 highest and 100 lowest level samples, which were further compared for levels of each gene. Results are shown as mean values ± SD and asterisks (∗) indicate significant differences from the low expression group (*P < 0.05, **P < 0.01, ***P < 0.001). **f** A putative mechanism for the roles of ribosomal dysfunction-associated PRDM1 in CRC stemness and cell survival.

The link of PRDM1 to cancer stemness was also verified in the ribosome-inactivated intestinal cancer. In the experimental intestinal tumor model of APC^Min/+^ mice, expression of Sox-9 and PRDM1 were significantly elevated in murine adenoma by gastrointestinal ribosomal dysfunction (Fig. 8d). Additionally, the involvement of PRDM1 in CRC stemness was tested in tumor spheroids. Ribosomal dysfunction increased the marker levels of intestinal cancer cell stemness (CD133-positives) in PRDM1-dependent way in cancer spheroids under the stress of ribosomal inactivation (Fig. 8e). Moreover, ribosomal dysfunction enhanced *CD133* expression in cancer spheroid cells, which was also mediated by PRDM1 (Fig. 8f). In order to get molecular signaling evidence in tumor cell stemness, the genome-wide microarray data of human CRC cells shown in Fig. 5e was revisited. In particular, Wnt signal-linked stemness markers were identified, which was verified in the ribosome-inactivated cancer spheroids (Fig. 9a). Notably, expression patterns of several Wnt-related signaling molecules such as Frizzled (FZD) proteins and Wnt ligands were disrupted by ribosomal dysfunction in PRDM1-dependent manners (Fig. 9a). Specifically, PRDM1-linked signals were positively associated with non-canonical Wnt-related genes (*Wnt5A* or *Frizzled (FZD) 4*) while suppressing canonical components (*Wnt6* or *FZD1*). Based on these results, it was thus assumed that non-canonical Wnt signaling-linked stemness is involved in PRDM1-linked tumor cell survival. TNP470, a non-canonical Wnt signaling inhibitor significantly reduced tumor cell survival from 5-FU under the ribosome-inactivating stress whereas canonical Wnt inhibition was marginally influential (Fig. 9b). Next, we addressed the signaling mechanism of PRDM1-associated differential regulation of chemoresistance. Ribosome-inactivated cells displayed increased levels of phospho-GSK3β (pGSK3β), an inactive form of GSK3β indicating active Wnt/β-catenin signaling (Fig. 9c). In addition, levels of RhoA protein and phospho-JNK (pJNK), indicating Wnt/PCP signaling activation, were also elevated by ribosomal dysfunction. Both RIS-activated canonical and non-canonical Wnt-linked signaling pathways were dependent on PRDM1. Furthermore. PRDM1-regulated transcriptional machinery was further investigated using the genome-wide microarray data of human CRC cells. In particular, caudal type homeobox transcription factor 2 (CDX2) was identified as a downstream target of PRDM1. CDX2 is known to be involved in differentiation of the intestinal stem cells into enterocytes^36^ and its deficiency is closely linked to increased risk of CRC^37^. PRDM1-deficient cancer cells displayed marginal expression of CDX2 in response to ribosomal dysfunction, indicating a positive regulation of CDX2 by PRDM1. In spite of suppressed PRDM1, overexpression of CDX2 restored levels of Wnt/PCP signaling molecules (RhoA and pJNK) while attenuating Wnt/β-catenin signaling activation (pGSK3β) (Fig. 9c). This differential regulation of Wnt signaling molecules during ribosome-inactivating stress was similar to that in clinical transcriptomic profile of CRC from the TCGA dataset (*n* = 440, COAD). High *PRDM1* group displayed enhanced levels of *Wnt5A, FZD4, RhoA*, and *ROCK1*, which are mainly involved in the Wnt/PCP signaling activation (Fig. 9d). In contrast, subjects with high PRDM1 had low expressions of Wnt/β-catenin positive regulators (*MYC* and *AXIN2*) and high levels of Wnt/β-catenin negative regulators (*APC* and *Dickkopf3 (DKK3)*) (Fig. 9e). Therefore, PRDM1 expression was positively associated with Wnt/PCP rather than canonical Wnt/β-catenin pathways in patents with CRC. Taken all, ribosomal dysfunction-responsive PRDM1-IGF signaling contributed to cancer cell stemness and subsequent tumor cell survival from anticancer actions via preferential regulation of non-canonical Wnt-linked pathways (Fig. 9f).

## Discussion

Since a large proportion of CRC etiologies are derived from stress responses, stress-induced PRDM1 was particularly crucial in tumor cell behavior in the harsh environment. Stress-driven ribosomal dysfunction elevated intestinal PRDM1 which was notably elevated in the progenitor-rich crypt parts of the gut and adenomas. Moreover, CRC spheroids displayed more cancer stemness markers in response to ribosomal dysfunction in PRDM1-IGF-dependent ways. Ribosome-inactivated CRC cells survived more from chemotherapeutic treatment via PRDM1-IGF-mediated regulation of non-canonical Wnt-linked cancer cell stemness, all of which provided mechanistic evidence of chemoresistance of CRC. In addition to the tumor growth promotion, stress-linked PRDM1 may play crucial roles in cancer cell survival from adverse environment including anticancer actions of chemotherapeutics. These beneficial actions were due to cancer stemness-associated cellular transformation and enhanced resistance to the cytotoxicity rather than the simply growth promoting action of PRDM1. Although it would be hard to address the clinical evidence in stress-linked CRC progression, stress response-induced PRDM1 provided important molecular clues for the link. The ribosomal dysfunction by stressors from the internal and external niche of the gut makes etiologic contribution to the sporadic CRC. Although the etiologies of the sporadic CRC are not well-known, the present clinical and biological verifications suggest potent roles of the gut ribosomal dysfunction in the RPDM1-linked tumor responses. Patient-based measurement of ribosomal dysfunction or its traces would be a better chance of new challenge of this study.

In addition to the PRDM1, some other types of PRDM are known as prognostic markers of epithelial cancers. For instance, upregulated expression of PRDM14 is positively associated with breast cancer cell growth via a reduction in their sensitivity to chemotherapeutic drugs^38^, while PRDM5 is epigenetically silenced in tumor tissues originating from the breast, liver, ovary, cervix, and gastrointestinal tract^39,40^. However, investigation of the activated B cell-like diffuse large B-cell lymphoma (DLBCL) demonstrated the presence of variable percentages of PRDM1-positive tumor cells in approximately half of the DLBCL cases and showed that patients with higher PRDM1 expression are significantly correlated with shorter failure-free survival^41^, which raises the possibility that higher PRDM1 expression is positively linked to tumor progression. Although the basal levels of PRDM1 in the enterocytes are relatively low, the present study demonstrated that stress-induced PRDM1 increased the survival-promoting actions and hampered the adverse action of chemotherapeutic agents. Although ribosomal dysfunction in itself could retard the growth of cancer cells in vitro^42–44^, the surviving CRC cells were resistant to cancer treatment, providing the source leading to cancer progression in the long run. In order words, chronic and frequent ribosomal dysfunction may allow cancer cells to adapt to the cytotoxic environment and survive via PRDM1, all of which would aggravate the cancer treatment as the malignant latency.

Mechanistically, ribosomal stress experience-induced PRDM1 positively regulated IGF receptor-linked pro-survival signals, counteracting the tumor-suppressive action of chemotherapeutics. IGFBP3 was suggested as a potent mediator between the pro-apoptotic action and survival response in the present study. Functions of IGFBP3 were initially defined as the main carrier of IGF-1 in circulation and primary protectors of free IGF-I for slower release and low availability of IGF to interact with the IGF receptor^45^. The IGFs promote cell survival and proliferation by binding to the IGF receptor; therefore, IGFBP3 is considered to regulate growth by limiting the IGF receptor-linked signal cascade^46^. In addition to effects on growth promotion, PRDM1 was also involved in cancer cell stemness and subsequent chemoresistance in RIS-aggravated CRC cells in the non-canonical Wnt signaling pathway. Although canonical Wnt signal generally acts by modulating the self-renewal activity of cancer stem cells, the effects of non-canonical Wnt5a on tumorigenesis are relatively diverse depending on cancer types and stages. In particular, Wnt5a is frequently epigenetically inactivated in CRC, showing tumor-suppressive activity through counteraction of the canonical Wnt/beta-catenin signaling pathway and CRC metastasis^47,48^. However, our results indicate that PRDM1-induced non-canonical Wnt5a was linked to chemoresistance in the ribosome-inactivated cancer cells. Consistent with our study, Wnt5a has been suggested as a biomarker of poor prognosis and chemoresistance in patients with epithelial tumors such as breast and ovarian cancer^49,50^. Mechanistically, Wnt5a could promote resistance to anticancer agents through Wnt/Protein kinase C-mediated survival signals^51^ or induction of ATP-binding cassette sub-family B member 1, a xenobiotic exporter, through activation of the protein kinase A (PKA)/β-catenin pathway^52^. In our model, the expression of non-canonical Wnt signaling mediators including Wnt5a and FZD4 were positively regulated by PRDM1, which could be a potential intervention target in the sporadic CRC. However, recently published report suggested that PRDM1 silences stem cell-related genes including the canonical Wnt signals^12^. Instead, we suggest that PRDM1-linked modulation of the balance between canonical and non-canonical Wnt signaling in the colon cancer. While suppressing the canonical pathways, PRDM1 was shown to elevate the non-canonical Wnt/planar cell polarity (PCP) signaling transduction in the ribosomal stress-associated colon cancer model. Based on these evidence, development of clinically applicable molecular interventions is warranted to overcome stress-altered cancer stemness and resistance to chemotherapy against CRC. The present study provides a promising model of gene-environment crosstalk via ribosome and mechanistic evidence for its potent impact on the chemoresponses and cancer stemness. It gives breakthroughs in modulation of the PRDM1-involved signaling network from the stress sentinel responsible for cancer cell survival and the malignant latency in response to cancer treatment.

## Methods

### Human colon cancer samples

Human colon tumor and adjacent normal tissues were obtained from the Korea Biobank Network of Pusan National University Hospital (Busan, South Korea). All biopsies were obtained with informed consent under Institutional Review Board-approved protocols (PNU IRB/2016_97_BR). Clinical characteristics of thirty patients included age (42–85 years old), gender (20 male, 10 female) and cancer stage (7 patients at state I, 9 at stage II, 12 at stage III, and 2 at stage IV).

### Survival and transcriptomic analysis using public datasets

Analysis of PRDM1 transcript was from tissues in the patients with the colon cancer (*Marisa*’s, GEO ID: gse39582, *n* = 557), colorectal cancer (*Sveen*’s, GEO ID: gse24551, *n* = 320), or colon adenocarcinoma (TCGA-COAD, *n* = 155) generated by the Cancer Genome Atlas (TCGA) Research Network: http://cancergenome.nih.gov/. KEGG-based functional analysis in association with PRDM1 was performed using datasets of CRC patients (TCGA-COAD (*n* = 286), and SieverSmith (gse14333 and gse17538, *n* = 355)). Using TCGA-based datasets, the association between PRDM1 and other genes in human CRC was analyzed.

### Cell culture and chemical treatment

HCT-8, DLD1, LOVO, SW480, and HT29 cells were purchased from the American Type Culture Collection (ATCC, Manassas, VA, USA). HCT-116, a human colon cancer cell line, and the isogenic HCT-116 p53(−/−) cell line were kindly provided by Bert Vogelstein at Johns Hopkins University (Baltimore, MD, USA). Cells were maintained in RPMI 1640 medium (Welgene, Daegu, Korea) supplemented with 10% [v/v] heat-inactivated FBS (Welgene), 25 mM HEPES (LPS solution, Daejeon, Korea), 50 U/ml penicillin, and 50 mg/ml streptomycin (Welgene) in a 5% CO_2_ humidified incubator at 37 °C. Platinum-A Retroviral Packaging Cells were purchased from Cell Biolabs (San Diego, CA, USA) and maintained in DMEM medium (Welgene, Gyeongsan, Korea) supplemented with 10% [v/v] heat-inactivated fetal bovine serum (FBS) (Welgene), 50 U/ml penicillin, and 50 mg/ml streptomycin (Welgene) in a 5% CO_2_ humidified incubator at 37 °C. Cell number was assessed by trypan blue (Sigma-Aldrich, St. Louis, MO, USA) dye exclusion using a hemacytometer. For the RIS exposure levels, the half-inhibitory dose of the total protein synthesis was determined in HCT-8 cells (50 ng/ml RIS-1 (anisomycin), 5 ng/ml RIS-2 (T-2), and 500 ng/ml RIS-3 (DON)). T-2 toxin (≥98% HPLC) isolated from *Fusarium sp*., anisomycin (≥98% HPLC) isolated from *Streptomyces griseolus*, deoxynivalenol (≥97.6% HPLC) isolated from *Fusarium graminearum* and other chemicals were purchased from Sigma-Aldrich.

### Protein synthesis analysis

HCT-8 cells were exposed to serial concentrations of RIS-1, RIS-2 or RIS-3 for 24 h, and changes in total protein concentration per cell (protein synthesis) for 24 h were measured. Levels of total protein synthesis from 0 to 24 h in the absence or presence of the RIS were compared, and the percentiles of protein synthesis inhibition levels per cell were calculated and statistically analyzed by linear regression. For the RIS quantitation, 90% inhibitory dose (IC_90_) of the total protein synthesis was determined in HCT-8 cells (250 ng/ml RIS-1 (anisomycin), 25 ng/ml RIS-2 (T-2), and 1000 ng/ml RIS-3 (DON)). In most cellular experiments, cells were treated with IC_90_ of RIS. In the harsh condition of the chemotherapeutic insult, cancer cells were exposed to IC_80_ of RIS.

### Construction of plasmid

CMV-driven short hairpin RNA (shRNA) was constructed by inserting shRNA into pSilencer 4.1-CMV-neo vector (Ambion, Austin, TX, USA). The control vector, shRNA of PRDM1, PKR, or IGFBP3 were denoted as shControl, shPRDM1, shPKR, shIGFBP3, or shCDX2, respectively. Inserts of PRDM1, PKR, IGFBP3, and CDX2 shRNA targeted 5′-GAT CTG ACC CGA ATC AAT G-3′, 5′-GCG AGA AAC TAG ACA AAG T-3′, 5′-GCA CAG ATA CCC AGA ACT T-3′, and 5′-ACA AAT ATC GAG TGG TGT A-3′, respectively. Flag-tagged PRDM1 gene expression construct was kindly provided by Dr. Tom Maniatis (Columbia University, New York, NY, USA). The Flag-tagged PRDM1 construct was amplified with the following primers: 5′-GAA TTC GGG ATG GCG ATG TTG GAT ATT TGC TTG GAA AAA CG-3′ (forward) and 5′-GAA TTC TTA AGG ATC CAT TGG TTC AAC TGT TT-3′ (reverse). The resulting 2477 bp construct for PRDM1 was cloned by excision at the EcoRI sites, followed by transfer into the PMX-IRES-GFP vector (Invitrogen, Carlsbad, CA, USA) using T4 DNA ligase (NEB, Beverly, MA, USA) to generate the PRDM1 vector. CDX2 cDNA was cloned by excision at the KpnI and XbaI site at pcDNA3.1-hygromycin as the template vector (ThermoFisher Scientific (#V87020, Sunnyvale, CA, USA).

### Transient and stable transfection

Cells were transfected with a mixture of plasmids using jetPRIME (Polyplus Transfection, New York, NY, USA) or OmicsFect (Omicsbio, Taipei, Taiwan) according to the manufacturer’s protocol and previous reports^26,53,54^. Transfection efficiency was confirmed by expression of a pMX-GFP vector (Cell Biolabs). To induce transient expression of shp53 or MEK DN, cells were transfected using jetPRIME. At 4 h after transfection with jetPRIME, the medium was changed and the cells were incubated for another 48 h. To create shPRDM1 or shIGFBP3 stable cell lines, cells were transfected using jetPRIME (Polyplus). After 48 h, transfected cells were subjected to selection for stable integrants by exposure to 700 μg/ml G418 (Life Technologies, Carlsbad, CA, USA) in complete medium containing 10% FBS. Selection was continued until monolayer colonies formed. The transfectants were then maintained in medium supplemented with 10% FBS and 350 μg/ml G418. PRDM1-PMX-IRES-GFP was transfected with a mixture of plasmids using OmicsFect (Omicsbio) according to the manufacturer’s protocol in PLAT-A cells, and the recombinant retroviral supernatant medium was collected after additional 24 h of incubation. HCT-8 cells were cultured in the retroviral supernatant medium for 24 h, after which PRDM1-expressing HCT-8 cells were selected by sorting the cells based on GFP fluorescence using a FACS Aria sorter (BD Bioscience, San Jose, CA, USA).

### Western blot analysis

Levels of protein expression were compared by western immunoblot analysis according to the previous reports^26,53,54^. Briefly, cells were washed with ice-cold phosphate buffer, lysed in boiling lysis buffer (1% [w/v] SDS, 1.0 mM sodium orthovanadate, and 10 mM Tris [pH 7.4]), and sonicated for 5 s. Lysates containing proteins were quantified using a BCA protein assay kit (Pierce, Rockford, IL, USA), after which 50 µg of protein was separated by BioRad mini gel electrophoresis (BioRad, Hercules, CA, USA). Proteins were transferred onto PVDF membrane (Pall Corporation, New York, NY, USA), after which the blots were blocked for 1 h with 5% skimmed milk in Tris-buffered saline plus Tween 0.1% (TBST) and probed with a 1:1000 dilution of each primary antibody (rabbit polyclonal anti-β-actin, mouse monoclonal anti-p53, goat polyclonal anti-Gdf15, rabbit polyclonal anti-RhoA, mouse monoclonal anti-PRDM1, mouse monoclonal anti-pERK1/2, mouse monoclonal anti-pJNK1/2 antibody (Santa Cruz Biotechnology, Dallas, TX, USA), rabbit polyclonal anti-pGSK3b (S9), rabbit polyclonal anti-CDX2 (ABclonal, Wuhan, China), or rabbit polyclonal anti-IGFBP3 (Bioss Antibodies, Woburn, MA, USA)) for additional 2 h at room temperature or overnight at 4 °C. After washing three times with TBST, blots were incubated with horseradish-conjugated secondary antibody for 2 h, then washed with TBST three times. Finally, protein was detected by pico enhanced peroxidase detection (ELPIS Biotech, Daejon, Korea). All original uncut blots are shown in Supplementary Fig. 4.

### Reverse transcription (RT) conventional PCR and real-time PCR

RNA was extracted using RiboEX (GeneAll Biotechnology, Seoul, Korea) according to the manufacturer’s instructions and following procedures are based on the previous report^55^. RNA (100 ng) from each sample was then transcribed to cDNA using Prime Moloney murine leukemia virus reverse transcriptase (Genetbio, Nonsan, Korea), after which samples were amplified using N-Taq DNA polymerase (Enzynomics, Daejeon, Korea) in a MyCycler thermal cycler (BioRad). The amplification parameters were as follows: denaturation at 95 °C for 2 min, followed by varying numbers of cycles of denaturation at 95 °C for 30 s, annealing at 58 °C for 30 s, and elongation at 72 °C for 30 s. The following primers were used for PCR: human PRDM1, 5′-GCA TGA ATG GCA TCA ACA AC-3′ and 5′-GCC GGT CAT GTT TCT TTT GT-3′; human RAB25, 5′-CCA TCA CCT CGG CGT ACT AT-3′ and 5′-GTC CGG ATG CTG TTC TGT CT-3′; human IGFBP3, 5′-GGT GTC TGA TCC CAA GTT CC-3′ and 5′-AGG CTG CCC ATA CTT ATC CA-3′; human IGF2, 5′-AAG TCG ATG CTG GTG CTT CT-3′ and 5′-GGA CTG CTT CCA GGT GTC AT-3′; and human IGF1R, 5′-TGA GGA TCA GCG AGA ATG TG-3′ and 5′-CAG AGG CAT ACA GCA CTC CA-3′; human CD133, 5′-TGC TGC TTG TGG AAT AGA CAG AAT G-3′ and 5′-AGG AAG GAC TCG TTG CTG GTG AA-3′; human MITF, 5′-CTG GCC AAA GAG AGG CAG AA-3′ and 5′-ATG CTG AAG GAG GTC TTG GC-3′; human FZD1, 5′-TCC ATC TGG TGG GTG ATC CT-3′ and 5′-CTT CTC GGT CTT GGT GGC AT-3′; human FZD4, 5′-GAA GAGGCA GCA GAA CCT GT-3′ and 5′-GTA AAG AGG GGA GCC ACC AC-3′; human WNT6, 5′-GCA ACA GGA CAT TCG GGA GA-3′ and 5′-GCC TCG TTG TTG TGC AGT TG-3′; and human WNT5a, 5′-CTT TGG GGA TGG CTG GAA GT-3′ and 5′-ATC TGC ATC ACC CTG CCA AA-3′. Aliquots of each PCR product were subjected to 1% (w/v) agarose gel electrophoresis and visualized by ethidium bromide (EtBr, Sigma-Aldrich) staining. For real-time PCR, FAM was conjugated to the 5′ ends of the probes as the fluorescent reporter dye to detect amplified cDNA. Analyses were conducted using an iCycler thermal cycler (BioRad) to subject the samples to the following conditions: denaturation at 94 °C for 2 min, followed by 40 cycles of denaturation at 98 °C for 10 s, annealing at 59 °C for 30 s, and elongation at 72 °C for 45 s. All analyses were conducted in triplicate and relative quantification of gene expression was accomplished using the comparative threshold cycle (CT) method. The CT value is defined as the point at which a statistically significant increase in fluorescence has occurred. The number of PCR cycles (CT) required for FAM intensity to exceed a threshold immediately above the background was calculated for test and reference reactions. In all experiments, GAPDH was used as the endogenous control.

### Quantification of apoptosis by flow cytometry

Apoptotic cells were quantified using an Annexin V-FITC detection kit (BD Bioscience) and detected by flow cytometry according to the previous reports^33,56^. Briefly, RIS-exposed cells were washed with PBS and harvested cells with trypsin were washed with PBS and resuspended with 100 μl of Annexin V-binding buffer. Cells were incubated with Annexin V (BD Biosciences, 1:40) and 100 μg/ml PI at room temperature for 15 min. Finally, the stained cells were subjected to flow cytometry (FACS Calibur, Becton Dickinson, San Jose, CA, USA) and analyzed by the CellQuest software (Becton Dickinson Co.).

### Murine tumorigenesis model

Animal care and experiments were ethically performed according to the guidelines of the Pusan National University Institutional Animal Care and Use Committee (PNUIACUC; Approval Number PNU-2017-1555). APC^min/+^ mice were provided by Dr. Kang-Yell Choi (Yonsei University, Seoul, Korea). A total of 25 mg anisomycin (Sigma-Aldrich) per average body weight of mouse (kg) and an equal volume of PBS were directly administered into 8-week-old APC^min/+^ and C57BL6 mice by oral gavage. After fostering for 3 days, the intestines of sacrificed mice were analyzed by immunohistochemistry.

### Murine allograft of CRC cells

All procedures are based on the previous report^33^. CMT-93, a C57BL/6 mouse colon cancer cell line, was purchased from the ATCC (Manassas, VA, USA) and maintained in DMEM (Welgene) supplemented with 10% (v/v) heat-inactivated FBS (Welgene), 50 units/ml penicillin, and 50 μg/ml streptomycin (Welgene) in a 5% CO_2_ humidified incubator at 37 °C. CMT-93 pre-exposed to 500 ng/ml of DON for 24 h was dissociated into single cells with trypsin. Next, 5 × 10^6^ cells were resuspended with 200 μl of PBS and injected subcutaneously into the dorsal side of 14-week-old male C57BL/6 mice (Hyochang Science, Daegu, South Korea). Seven days later, 100 mg/ml 5-FU was intraperitoneally injected, and the tumor tissues were surgically excised 24 h later.

### Tumor xenograft assay

For tumor inoculation, 1.5 × 10^6^, control, shPRDM1- or PRDM1-overexpressing cells were subcutaneously injected into each flank of 5-week-old BALB/C nu/nu female mice (Orient Bio, Seongnam, Korea). Control cells were injected into the left flank and HCT-8, shPRDM1- or PRDM1-overexpressing cells were injected into the right flank. Tumor size was measured with calipers 15–30 days after injection. Tumor dimensions were converted to the tumor volume as follows: length × width × height = mm^3^. This study was carried out in accordance with the Helsinki Declaration after approval was received from the Institutional Animal Care and Use Committee of Pusan National University Hospital (IRB No. PNIU-2012-0083).

### Immunohistochemistry

All procedures are based on the previous reports^57–59^. Tumor samples were dehydrated, embedded in paraffin, and cut into 5 μm sections for immunohistochemistry (IHC) analysis. Prior to being subjected to immunostaining, formalin-fixed paraffin-embedded tumors from mouse flanks were cut (5 μm), deparaffinized, and rehydrated. Tissue slides were heated in 10 mM sodium acetate (pH 9.0) for 5 min at 121 °C for antigen retrieval, then bathed in a 3% H_2_O_2_-PBS solution for 15 min at room temperature in the dark to quench endogenous peroxidase. After samples were washed with 0.5% Tris-HCl-Tween, tissue sections (5 μm) were blocked with 3% bovine serum albumin (BSA, Bovogen Biologicals, Melbourne, Australia) in PBS, incubated in a 1:200 dilution of each primary antibodies (mouse monoclonal anti-p53, goat polyclonal anti-Gdf15, mouse monoclonal anti-PRDM1, rabbit polyclonal anti-SOX9, or mouse monoclonal anti-p-ERK antibody (Santa Cruz Biotechnology); rabbit polyclonal anti-total ERK1/2 antibody (Cell Signaling Technology, Danvers, MA, USA); rabbit polyclonal anti-IGFBP3 antibody (Bioss Antibodies, Woburn, MA, USA) overnight at 4 °C, and repeatedly washed using PBS. Samples were incubated with horseradish peroxidase-conjugated secondary antibody for 2 h at room temperature, after which they were subjected to repeated washing with PBS. The bound antibodies were then identified using freshly prepared substrate buffer (0.05% diaminobenzidine [DAB; Sigma-Aldrich], 0.015% H_2_O_2_ in PBS) for 2 min. After a final wash in PBS and distilled water, the slides were counterstained with a 50% dilution of hematoxylin (Santa Cruz Biotechnology) for 1 min, then dehydrated in graded alcohols (50%, 70%, 80%, 90%, 95%, 100%, and 100%). Sections were subsequently examined at various magnifications using an Axio Imager M2 (Carl Zeiss MicroImaging, GmbH, Oberkochen, Germany). To analyze the proportion of positive stained cells in the tissue, at least four representative areas were measured by computer-assisted analysis using Histo-quest software 4.0 (TissueGnostics GmbH, Vienna, Austria).

### Spheroid culture and immunocytochemistry of spheroids

HCT-8 cells (2.5 × 10^5^ per well) were seeded in the ultralow attachment 6-well plates (Corning Incorporated, Corning, NY, USA). Spheroid cells were cultivated for 6 days in the spheroid culture media (DMEM containing B27 (Life Technology, Waltham, MA, USA)), 20 ng/ml of bFGF (PEPROTECH, Rocky Hill, NJ, USA), 20 ng/ml of recombinant human EGF (Cell Signaling), and 1% antibiotics. To verify the competence of colonospheres, 6-day cultured spheroids were fixed with 4% paraformaldehyde containing 1% triton X-100 at 4 °C overnight, then fixed with 4% paraformaldehyde over 2 h. Spheroids were washed with PBS, blocked with 3% BSA, and then incubated with mouse anti-human CD44 (1:200, Cell signaling) at 4 °C overnight. The fixed spheroids were subsequently washed in PBS, incubated with Cy3-conjugated goat anti-mouse IgG (Abcam, Cambridge, UK) for 2 h at room temperature, washed in PBS, and counterstained with 100 ng/ml DAPI (absorbance at 405 nm, Sigma-Aldrich) in PBS for 10 min. Spheroids were washing with PBS 5 times for 8 min each, observed, and analyzed under a confocal laser scanning microscope (FV1000, Olympus, Nagano, Japan). To compare sphere sizes, about 200 spheroids per group were randomly selected, after which their perimeters were measured and statistically analyzed.

### Statistics and reproducibility

Statistical analyses were performed using GraphPad Prism v. 5.01 (La Jolla, CA, USA) according to the previous report^54^. For comparative analysis of two groups of data, Student’s *t* test was performed. For comparative analysis of multiple groups, data were subjected to analysis of variance (ANOVA) with Newman–Keuls method as a post hoc ANOVA assessment. For two gene correlation coefficient (R) determination in CRC-based datasets, Pearson’s correlation analysis was performed. All in vitro evaluations are representative of two or three independent experiments. Details of the number of biological replicates and the assays are given in each figure legends.

### Reporting summary

Further information on research design is available in the Nature Research Reporting Summary linked to this article.

## Supplementary information

Peer Review File

Supplementary Information

Descriptions of Additional Supplementary Files

Supplementary Data 1

Reporting Summary

## Data Availability

All data needed to evaluate the conclusions in the paper are present in the paper. The NCBI repository number of the microarray data in Fig. 5e is GEO ID: gse171475. Additional data related to this paper are available from the corresponding author on reasonable request. The Source data underlying plots shown in figures are provided in Supplementary Data 1.
