# Supplementary material for: Mucosal ribosomal stress-induced PRDM1 promotes chemoresistance via stemness regulation
Source: Commun Biol. 2021 May 10;4:543. doi: 10.1038/s42003-021-02078-1 (PMC8110964; doi:10.1038/s42003-021-02078-1)
Supplement: Supplementary file 2 — Supplementary Information [file 42003_2021_2078_MOESM2_ESM.pdf]

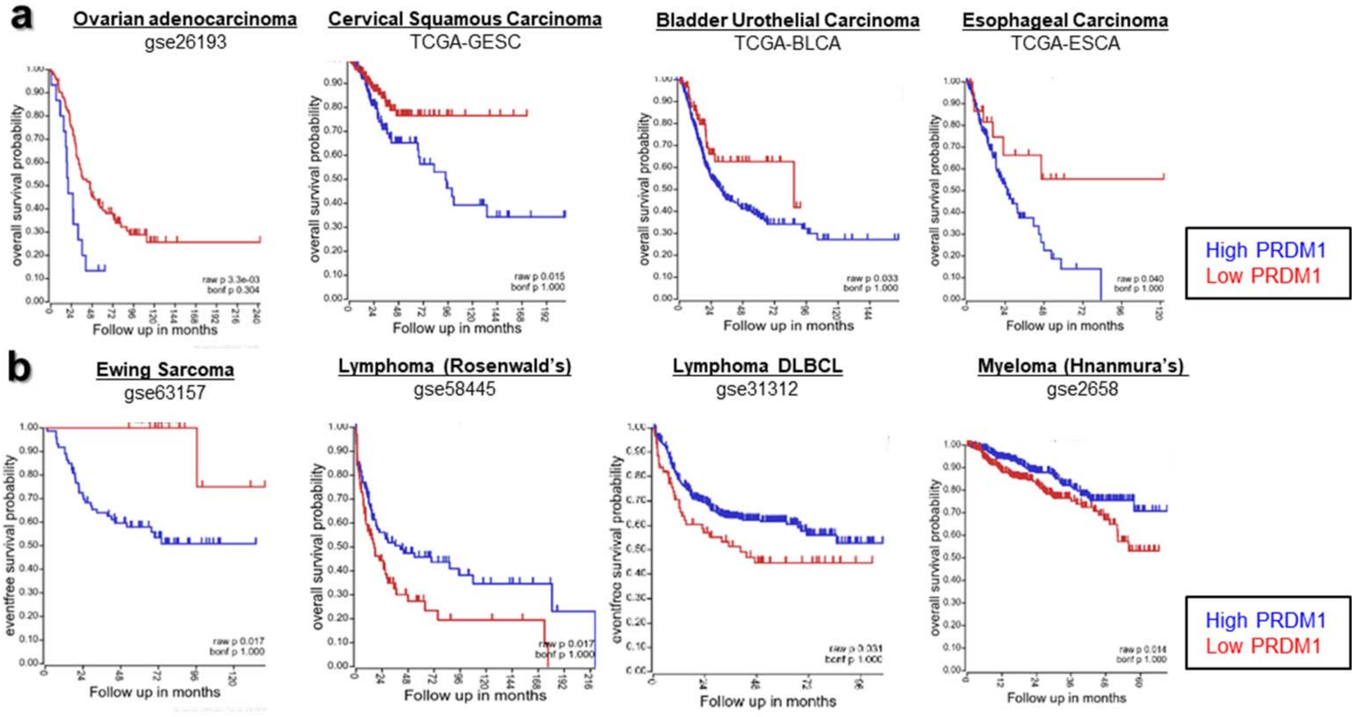

Supplementary figure 1. Histological category-based cancer patient survival in terms of PRDM1 levels. (a) Analysis of PRDM1 expression level in patients with ovarian adenocarcinoma (GEO ID: gse26193, n=107), cervical squamous cell carcinoma (TCGA-GESC, n=305), bladder urothelial carcinoma (TCGA-BLCA, n=408) and esophageal carcinoma (TCGA-ESCA, n=184). (b) Analysis of PRDM1 expression level in patients with Ewing Sarcoma (GEO ID: gse63157, n=85), lymphoma (GEO ID: gse58445, n=193), lymphoma DLBCL (GEO ID: gse31312, n=498) and myeloma (GEO ID: gse2658, n=542).

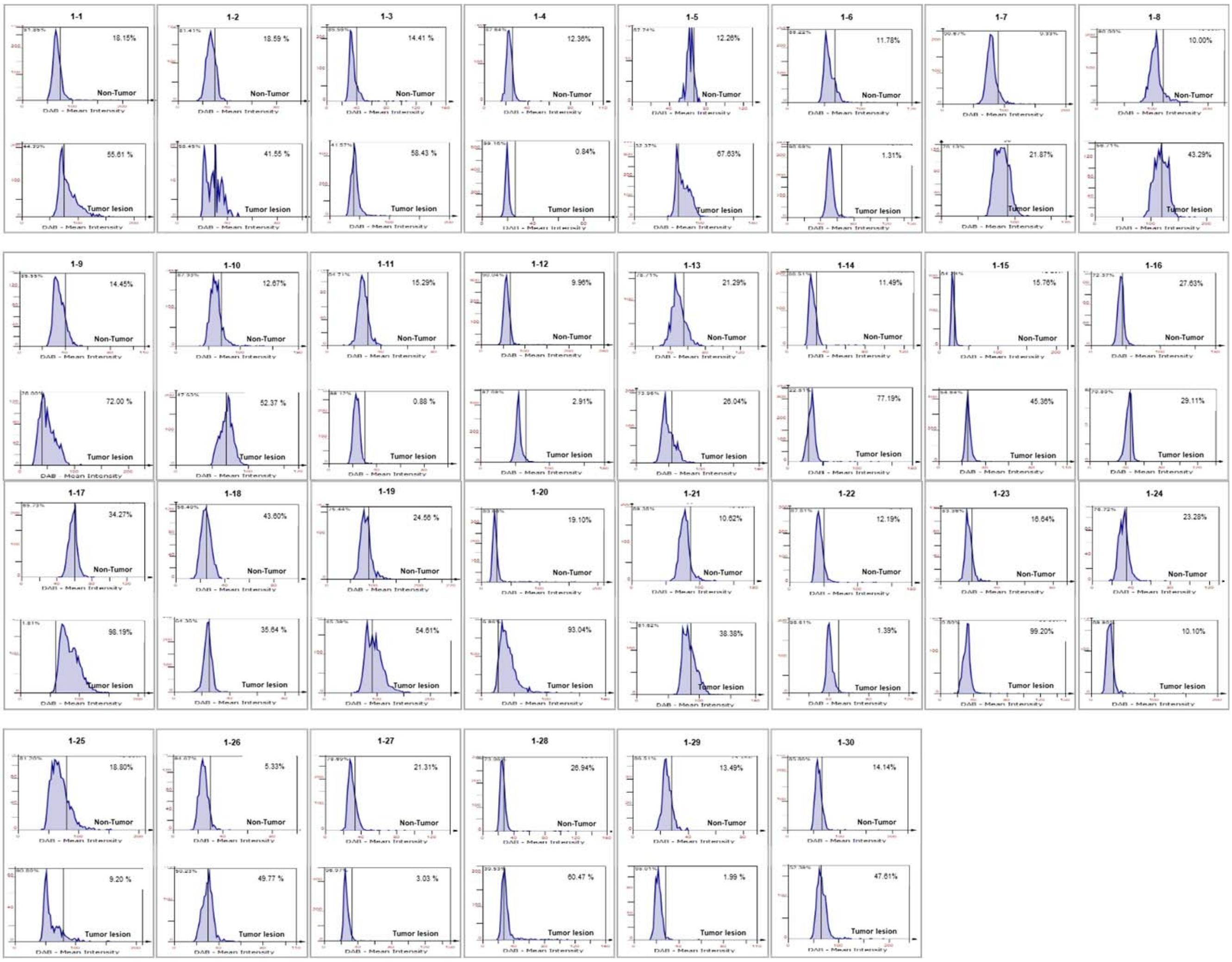

Supplementary figure 2. PRDM1 expression in patients with CRC. Each histogram couple represents quantitation of PRDM1 immunohistochemistry (IHC) of normally appearing parts (upper) and tumor lesions (lower) from each patient with CRC (n=30) using Histo-quest software 4.0.

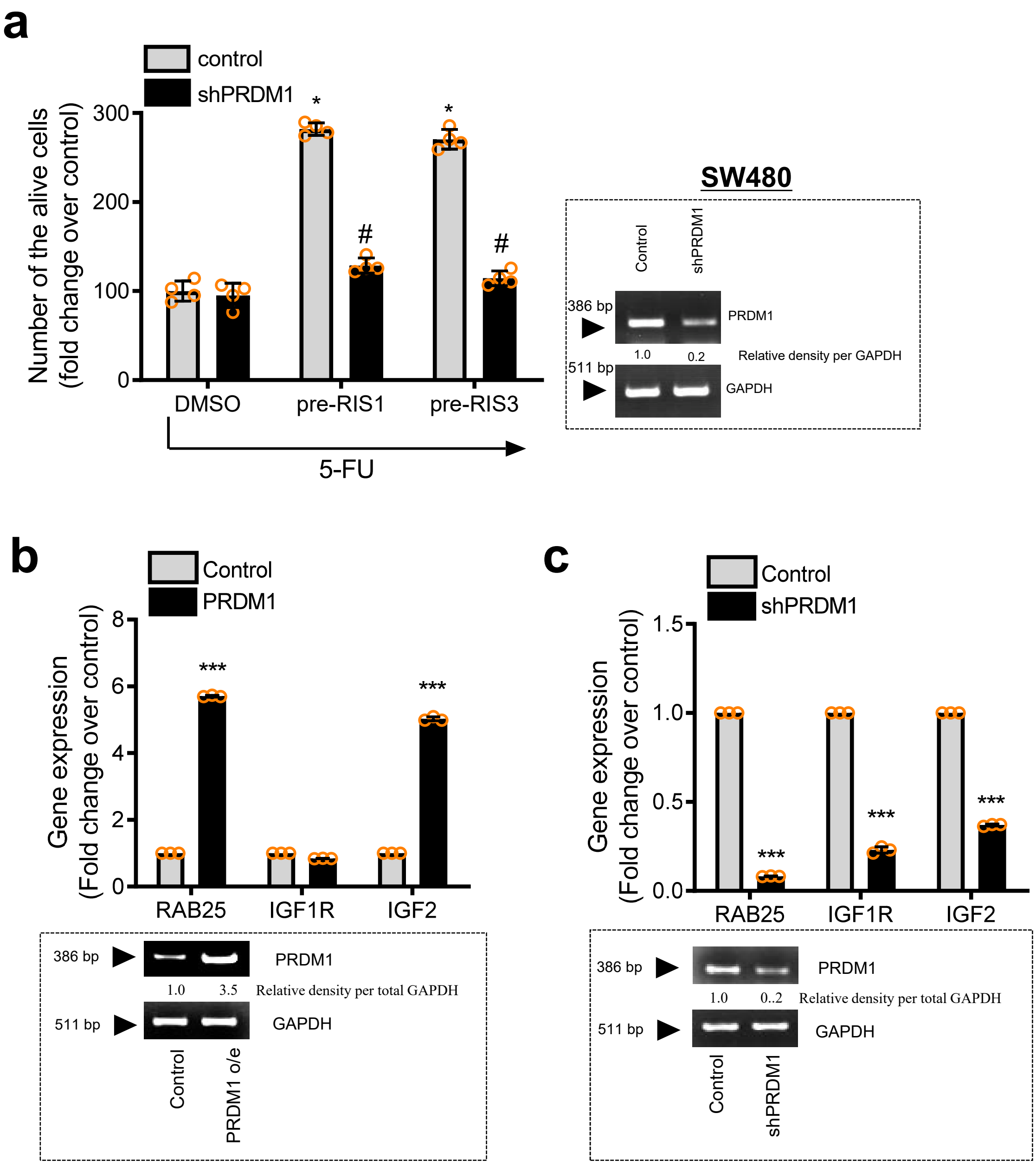

Supplementary figure 3. Roles of PRDM1 in CRC chemoresistance. (a) The negative control plasmid- or shPRDM1-expressing SW480 cells pre-exposed to ID_{80} of RIS-1 or RIS-3 for 24 hours were treated with 375 μM 5-FU for 48 hours. Live cells were counted. Results are shown as mean values ± SD. Asterisks represent a significant difference compared to each vehicle (DMSO) treatment group and the symbols (#) indicate a significant difference compared to each control cells (*P*<0.001). (b-c) The relative mRNA expressions in cell lines (control plasmid-, PRDM1 overexpression plasmid (b)-, or shPRDM1(C)-expressing HEK293) were quantified using RT real-time PCR. Results are shown as mean values ± SD and asterisks represent a significant difference relative to the control group (****P*<0.001).

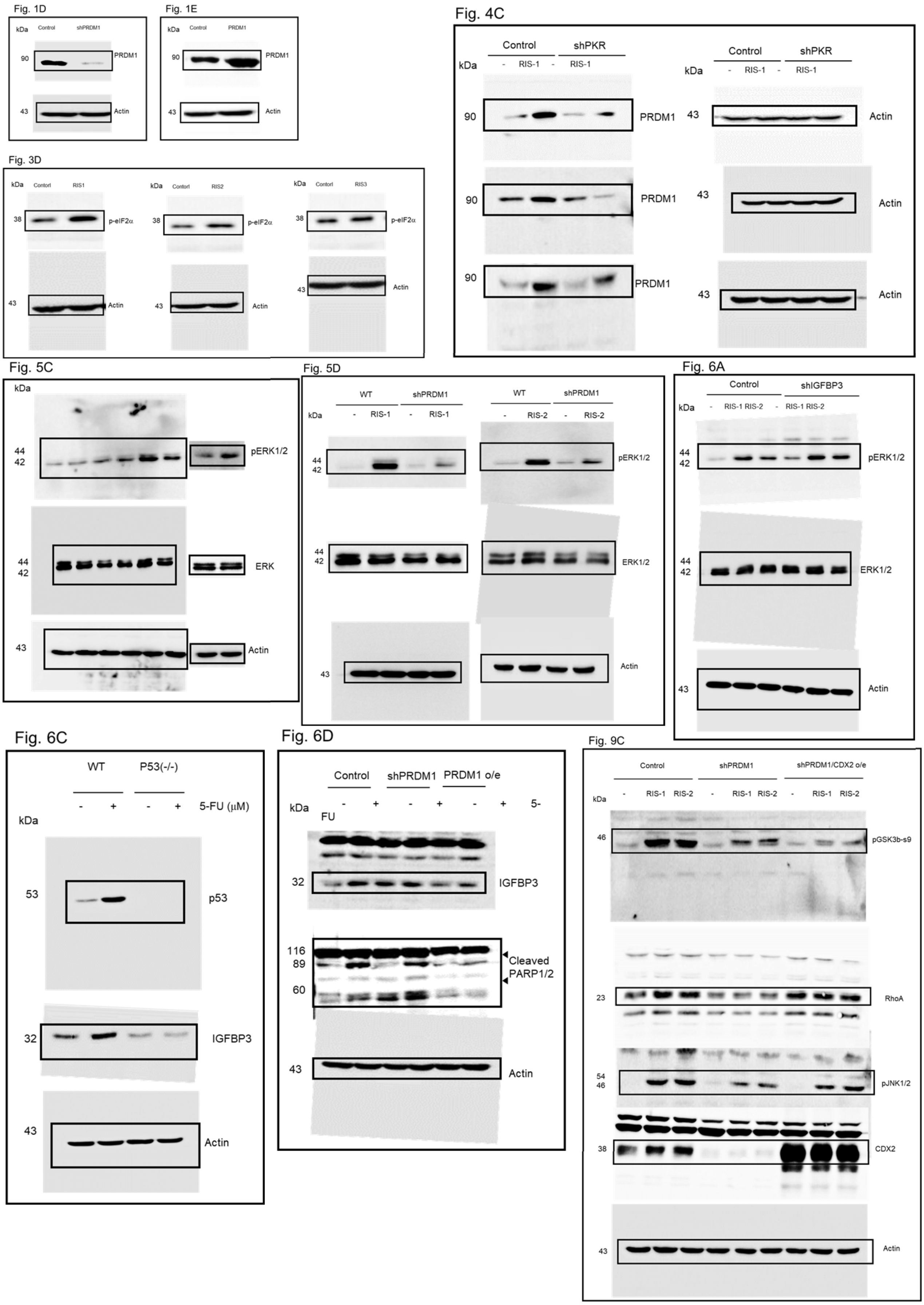

Supplementary figure 4. Original immunoblots for panels shown in Figures 1d, 1e, 3d, 4c, 5 c, 5d, 6a, 6c, 6d, and 9c.
