# Supplementary material for: Mucosal ribosomal stress-induced PRDM1 promotes chemoresistance via stemness regulation
Source: Commun Biol. 2021 May 10;4:543. doi: 10.1038/s42003-021-02078-1 (PMC8110964; doi:10.1038/s42003-021-02078-1)
Supplement: Supplementary file 3 — Descriptions of Additional Supplementary Files [file 42003_2021_2078_MOESM3_ESM.pdf]

Description of Additional Supplementary Files

Supplementary data 1
Description: Source data underlying plots shown in figures.
