# Supplementary material for: Mucosal ribosomal stress-induced PRDM1 promotes chemoresistance via stemness regulation
Source: Commun Biol. 2021 May 10;4:543. doi: 10.1038/s42003-021-02078-1 (PMC8110964; doi:10.1038/s42003-021-02078-1)
Supplement: Supplementary file 5 — Reporting Summary [file 42003_2021_2078_MOESM5_ESM.pdf]

nature research

Corresponding author(s): YUSEOK MOON

Last updated by author(s): MAR 19, 2021

Reporting Summary

Nature Research wishes to improve the reproducibility of the work that we publish. This form provides structure for consistency and transparency in reporting. For further information on Nature Research policies, see our Editorial Policies and the Editorial Policy Checklist.

Statistics

For all statistical analyses, confirm that the following items are present in the figure legend, table legend, main text, or Methods section.

n/a	Confirmed	
☐	☒	The exact sample size (*n*) for each experimental group/condition, given as a discrete number and unit of measurement
☐	☒	A statement on whether measurements were taken from distinct samples or whether the same sample was measured repeatedly
☐	☒	The statistical test(s) used AND whether they are one- or two-sided<br>*Only common tests should be described solely by name; describe more complex techniques in the Methods section.*
☒	☐	A description of all covariates tested
☐	☒	A description of any assumptions or corrections, such as tests of normality and adjustment for multiple comparisons
☐	☒	A full description of the statistical parameters including central tendency (e.g. means) or other basic estimates (e.g. regression coefficient) AND variation (e.g. standard deviation) or associated estimates of uncertainty (e.g. confidence intervals)
☐	☒	For null hypothesis testing, the test statistic (e.g. *F*, *t*, *r*) with confidence intervals, effect sizes, degrees of freedom and *P* value noted<br>*Give P values as exact values whenever suitable.*
☒	☐	For Bayesian analysis, information on the choice of priors and Markov chain Monte Carlo settings
☒	☐	For hierarchical and complex designs, identification of the appropriate level for tests and full reporting of outcomes
☐	☒	Estimates of effect sizes (e.g. Cohen's *d*, Pearson's *r*), indicating how they were calculated

Our web collection on statistics for biologists contains articles on many of the points above.

Software and code

Policy information about availability of computer code

Data collection	Public datasets (GSE39582, GSE24551, TCGA-COAD, GSE14333, GSE17538) were used.
Data analysis	Analyses were performed using GraphPad Prism v. 8.01 (La Jolla, CA, USA).

For manuscripts utilizing custom algorithms or software that are central to the research but not yet described in published literature, software must be made available to editors and reviewers. We strongly encourage code deposition in a community repository (e.g. GitHub). See the Nature Research guidelines for submitting code & software for further information.

Data

Policy information about availability of data

All manuscripts must include a data availability statement. This statement should provide the following information, where applicable:

- Accession codes, unique identifiers, or web links for publicly available datasets
- A list of figures that have associated raw data
- A description of any restrictions on data availability

All data needed to evaluate the conclusions in the paper are present in the paper. Additional data related to this paper are available from the corresponding author on reasonable request.

Field-specific reporting

Life sciences study design

All studies must disclose on these points even when the disclosure is negative.

Sample size	For comparative analysis of two groups of data, Student's t test was performed. For comparative analysis of multiple groups, data were subjected to analysis of variance (ANOVA) with Newman-Keuls method as a post hoc ANOVA assessment.
Data exclusions	N/A
Replication	All in vitro evaluations are representative of two or three independent experiments. Details of the number of biological replicates and the assays are given in each figure legends.
Randomization	Random allocation of experimental animals (mice) and cell cultures were applied in all experiments.
Blinding	Blinding was employed during data collection.

Reporting for specific materials, systems and methods

We require information from authors about some types of materials, experimental systems and methods used in many studies. Here, indicate whether each material, system or method listed is relevant to your study. If you are not sure if a list item applies to your research, read the appropriate section before selecting a response.

Materials & experimental systems

n/a	Involved in the study
☐	☒ Antibodies
☐	☒ Eukaryotic cell lines
☒	☐ Palaeontology and archaeology
☐	☒ Animals and other organisms
☒	☐ Human research participants
☒	☐ Clinical data
☒	☐ Dual use research of concern

Methods

n/a	Involved in the study
☒	☐ ChIP-seq
☐	☒ Flow cytometry
☒	☐ MRI-based neuroimaging

Antibodies

Antibodies used	The primary antibodies used were rabbit polyclonal anti-b-actin, mouse monoclonal anti-p53, goat polyclonal anti-Gdf15, rabbit polyclonal anti-RhoA, mouse monoclonal anti-PRDM1, mouse monoclonal anti-pERK1/2, mouse monoclonal anti-pJNK1/2 antibody (Santa Cruz Biotechnology, Dallas, TX, USA), rabbit polyclonal anti-pGSK3b (S9), rabbit polyclonal anti-CDX2 (ABclonal, Wuhan, China), or rabbit polyclonal anti-IGFBP3 (Bioss Antibodies, Woburn, MA, USA).
Validation	b-Actin 43 kDa https://www.scbt.com/ko/p/beta-actin-antibody-c4?requestFrom=search<br>p53 53 kDa https://www.citeab.com/antibodies/820814-sc-126-p53-antibody-do-1<br>Gdf15 40 kDa https://www.citeab.com/antibodies/795877-sc-10603-gdf-15-antibody-h-20<br>RhoA 24 kDa https://www.scbt.com/ko/p/rho-a-antibody-119<br>PRDM1 90 kDa https://www.scbt.com/p/blimp-1-antibody-n-20<br>pERK1/2 44/42 kDa https://www.scbt.com/ko/p/p-erk-antibody-e-4<br>PJNK1/2 46/54 kDa https://www.scbt.com/ko/p/p-erk-antibody-e-4<br>pGSK3b(S9) 46 kDa https://abclonal.com/catalog-antibodies/PhosphoGSK3S9RabbitmAb/AP1088<br>CDX2 33 kDa https://abclonal.com/catalog-antibodies/CDX2RabbitpAb/A0804<br>IGFBP3 32 kDahttps://www.biossusa.com/products/bs-1434r

Eukaryotic cell lines

Policy information about cell lines

Cell line source(s)	HCT-8, HEK293 and SW480 cells were purchased from American Type Culture Collection (ATCC, Manassas, VA, USA). HCT-116, a human colon cancer cell line, and the isogenic HCT-116 p53(-/-) cell line were kindly provided by Bert Vogelstein at Johns Hopkins University (Baltimore, MD, USA).
Authentication	All cell lines have authentication of the company (American Type Culture Collection (ATCC, Manassas, VA, USA).
Mycoplasma contamination	All cell lines test negative for mycoplasma contamination.
Commonly misidentified lines<br>(See ICLAC register)	N/A

Animals and other organisms

Policy information about studies involving animals; ARRIVE guidelines recommended for reporting animal research

Laboratory animals	C57BL/6 mice (6 weeks old, 16–18 g on average) were purchased from Hyochang science (Daegu, South Korea). BALB/C nu/nu female mice (5 weeks old, 15-17 g on average) were purchased from Orient Bio (Seongnam, South Korea).
Wild animals	N/A
Field-collected samples	Mice were acclimated for 14 days prior to experiments and maintained at 25°C in 45–55% relative humidity under 12 h light/dark cycles. Mice were housed three per cage and provided sufficient food and water in environmentally protected cages comprising a transparent polypropylene body and a stainless steel wire top cover.
Ethics oversight	Animal care and experimental procedures were conducted in accordance with our Institutional Animal Care and Use Committee's guidelines. This animal study was approved by the Pusan National University Institutional Animal Care and Use Committee (PNU-IACUC) (PNU-2017-1555, PNIU-2012-0083).

Note that full information on the approval of the study protocol must also be provided in the manuscript.

Flow Cytometry

Plots

Confirm that:

- [ ] The axis labels state the marker and fluorochrome used (e.g. CD4-FITC).
- [ ] The axis scales are clearly visible. Include numbers along axes only for bottom left plot of group (a 'group' is an analysis of identical markers).
- [ ] All plots are contour plots with outliers or pseudocolor plots.
- [x] A numerical value for number of cells or percentage (with statistics) is provided.

Methodology

Sample preparation	Treated cells were washed with PBS, after which collected cells were mixed in Annexin binding buffer (100 ul) and incubated with an Annexin V/PI double staining solution at room temperature for 15 min.
Instrument	FACS Calibur (BD Bioscience San Jose, CA, USA)
Software	CellQuest software (BD Bioscience, San Jose, CA, USA)
Cell population abundance	We were used the positive cells (positive for both Annexin V-FITC and PI) to set up compensation and quadrants.<br>> Annexin V negative - PI negative populations are healthy cells.<br>> Annexin V positive - PI negative populations represent cells in early apoptosis.<br>> Annexin V positive - PI positive staining indicate cells are in necrosis (post-apoptotic necrosis or late apoptosis).
Gating strategy	The gate was selected for all the cells excluding the debris.

- [ ] Tick this box to confirm that a figure exemplifying the gating strategy is provided in the Supplementary Information.
