# Supplementary material for: Mucosal ribosomal stress-induced PRDM1 promotes chemoresistance via stemness regulation
Source: Commun Biol. 2021 May 10;4:543. doi: 10.1038/s42003-021-02078-1 (PMC8110964; doi:10.1038/s42003-021-02078-1)
Supplement: Supplementary file 1 — Peer Review File [file 42003_2021_2078_MOESM1_ESM.pdf]

Reviewers' comments:

Reviewer #1 (Remarks to the Author):

In the present manuscript, Kim and Moon show that PR domain zinc finger protein (PRDM) 1 expression is positively correlated with a poor prognosis in colorectal cancer (CRC) patients. The authors suggest that "stress-driven ribosomal dysfunction" in CRC is responsible for upregulation of PRDM1, which promotes the CRC cell stemness, survival and resistance to 5-FU through upregulation of the IGF/non-canonical Wnt pathway. These findings are of general interest for the field and they may be of clinical value.

Although potentially interesting several major issues need to be addressed.

1) By using bioinformatic analysis of the CRC transcriptome data the authors reveal that that increased PRDM1 expression is linked to "the ribosome-associated events". Based on that, they conclude that increased levels of PRDM1 protein in CRC are caused by stress-driven ribosomal dysfunction. The authors should comment on the identity and functions of RNAs that may be involved in mediating "ribosomal dysfunction".

2) The authors report that stress-driven ribosomal dysfunction induced by treatment of CRC cells with with specific ribosome-inactivating stress (RIS) agents leads to PKR-dependent eIF2-ɑ phosphorylation, inhibition of general protein synthesis and enhancement of PRDM1 protein levels. However, they do not provide evidence that PRDM1 is upregulated by the same mechanism in human CRC in vivo (in the absence of treatment with RIS agents). For example, the authors may assess the status of eIF2ɑ phosphorylation in human CRC that have significantly elevated expression of PRDM1.

3) The authors report a strong inverse correlation between the expression of PRDM1 and p53 in tissues of the xenograft tumor model. What is the underlying molecular mechanism?

4) The authors show that enhanced PRDM1 levels in wild-type p53 HTC-8 cells upon treatment with RIS agents are responsible for resistance to 5-FU. Expression of wild-type p53 has been shown to greatly potentiate 5-FU-cytotoxicity. What is the contribution of low p53 levels to PRDM1-mediated resistance to 5-FU? What is the effect of silencing both wild-type p53 and PRDM1 on cytotoxicity of 5-FU in HTC-8 cells?

5) It is necessary to provide some insights into molecular mechanisms by which PRDM1 regulates the IGF/non-canonical Wnt pathway.

Reviewer #2 (Remarks to the Author):

The article of Juil Kim and Yuseoc Moon entitled "Ribosomal alert-induced PRDM1 spoils anticancer actions via stemness regulation" is an interesting and solid work. Before this article can be published in Communications Biology some corrections are needed.
My concerns are:
- The authors have to introduce abbreviation when they are first used; e.g. line 35.
- In line 131 the authors mention that they used 30 biopsies from CRC patients but only two cases are presented in Figure 2.
- In line 159 the text does not fit to the cited Figure. The authors must be much more accurate.
- Line 190: The mentioned Figure 3E right part must be presented as Table 1 and should be deleted from the Figure 3.
- Starting with line 432 the "Material and methods" part significantly decrease in quality. The authors have to increase the second part of this chapter significantly. Information about dilution of antibodies, or even the used antibodies (line 524) and the cities where the company is located are missing. Sometimes the authors don´t provide the company from which they have the reagents. The authors have to provide this information.
- What want the authors say with "m" in line 486? Normally "m" is an abbreviation for metres but

that makes no sense at all here.
- In lines 787-788 the authors have to be more specific.
- The authors have to organise the figures in a better way – this is the worst part of the whole manuscript (that has a high quality). In general Figures are overloaded and not well organized/structured. Figure 1: Blots are too small; part F and G should be fused together and combined as one part. Figure 2: Blots are too small; abbreviation in part A must be explained. Figure 3: Part C is too small.

The authors should think about the possibility to present their interesting findings in several better organized figures and not in such a crowded way. The only acceptable figure is S2.

Comments of Reviewer 1

In the present manuscript, Kim and Moon show that PR domain zinc finger protein (PRDM) 1 expression is positively correlated with a poor prognosis in colorectal cancer (CRC) patients. The authors suggest that "stress-driven ribosomal dysfunction" in CRC is responsible for upregulation of PRDM1, which promotes the CRC cell stemness, survival and resistance to 5-FU through upregulation of the IGF/non-canonical Wnt pathway. These findings are of general interest for the field and they may be of clinical value.

Although potentially interesting several major issues need to be addressed.

1. By using bioinformatic analysis of the CRC transcriptome data the authors reveal that that increased PRDM1 expression is linked to "the ribosome-associated events". Based on that, they conclude that increased levels of PRDM1 protein in CRC are caused by stress-driven ribosomal dysfunction. The authors should comment on the identity and functions of RNAs that may be involved in mediating "ribosomal dysfunction".

Response: As a reviewer pointed out, we added mention on identity and functions of ribosomal dysfunction via a key signaling such as *PKR signaling* and proved the correlation between PRDM1 and PKR as the key modulator of ribosomal stress responses using the clinical dataset of patients with CRC **(Fig. 3C)**. PKR expression was verified to be positively associated with PRDM1 expression in patients with CRC. "Line 74: *In particular, the ribosome stands sentinel to stress and stress-driven ribosomal dysfunction (ribosomal insult) triggers eukaryotic translation initiation factor 2 subunit α (eIF2α)-mediated global translational inhibition via protein kinase R (PKR), which is one of the primary integrated stress responses (ISR)* ……………… Line 149: *Clinical transcriptomic analysis indicated that CRC patients with high levels of EIF2AK2 (PKR) as the key modulator of ribosomal stress responses display increased levels of PRDM1 (Fig. 3C).*"

2. The authors report that stress-driven ribosomal dysfunction induced by treatment of CRC cells with specific ribosome-inactivating stress (RIS) agents leads to PKR-dependent eIF2-α phosphorylation, inhibition of general protein synthesis and enhancement of PRDM1 protein levels. However, they do not provide evidence that PRDM1 is upregulated by the same

mechanism in human CRC in vivo (in the absence of treatment with RIS agents). For example, the authors may assess the status of eIF2α phosphorylation in human CRC that have significantly elevated expression of PRDM1.

Response: As a reviewer pointed out, it is a great comment and we performed additional analysis for the link between PKR and PRDM1 expression in patients with IBD **(Fig. 3C)**, concluded as "Line 149: *Clinical transcriptomic analysis indicated that CRC patients with high levels of EIF2AK2 (PKR) as the key modulator of ribosomal stress responses display increased levels of PRDM1 (Fig. 3C).*"

3. The authors report a strong inverse correlation between the expression of PRDM1 and p53 in tissues of the xenograft tumor model. What is the underlying molecular mechanism?

Response: As a reviewer pointed out, we performed additional analyses by measuring p53-responsive reporter activity in response to PRDM1 levels **(Fig. 2C and 2D)** and concluded as "Line 124: *The inverse correlation between PRDM1 and p53 signaling was mechanistically verified using p53-responsive luciferase reporter system in the intestinal cancer cells. While genetic knockdown of PRDM1 elevated p53-responsive reporter activity, PRDM1 overexpression downregulated the reporter activity (Fig. 2C and 2D). Furthermore, p53-mediated reporter levels were assessed after treatment with 5-fluorouracil (5-FU) as a conventional chemotherapeutic agent, one of the first line anti-cancer drug against CRC. The chemotherapeutic agent increased p53-mediated reporter expression, which was also counteracted by PRDM1 (Fig. 2C and 2D). All of these evidences indicate that PRDM1 is a negative regulator of p53-responsive elements in both intact and 5-FU-treated cancer cells.*"

4. The authors show that enhanced PRDM1 levels in wild-type p53 HTC-8 cells upon treatment with RIS agents are responsible for resistance to 5-FU. Expression of wild-type p53 has been shown to greatly potentiate 5-FU-cytotoxicity. What is the contribution of low p53 levels to PRDM1-mediated resistance to 5-FU? What is the effect of silencing both wild-type p53 and PRDM1 on cytotoxicity of 5-FU in HTC-8 cells?

Response: It is an excellent comment. As a reviewer pointed out, we performed additional experiments using p53KO HCT-116 cells **(Fig. 5B).** The results were denoted as "Line 182:

Furthermore, p53 was evaluated as a potent component involved in PRDM1-mediated cellular responses since PRDM1 counteracted p53-responsive transcriptional activity (Fig. 2C and 2D). Genetic ablation of p53 increased ribosomal dysfunction-driven chemoresistance even when PRDM1 expression was suppressed (Fig. 5B). All evidences indicate that PRDM1 counteracts p53-mediated cytotoxicity of chemotherapeutic 5-FU in the ribosome-inactivated cancer cells."

- Additionally, we provided p53-downstream mechanism as "Line 230: *In contrast with survival-linked ERK1/2 signaling, IGFBP3 was positively regulated by apoptosis-associated p53 in response to 5-FU treatment (Fig. 6C). Consistent with the genome-wide microarray data of human CRC cells (Fig. 5E), PRDM1 counteracted IGFBP3 expression and chemotherapeutic agent-induced apoptosis (Fig. 6D). In particular, IGFBP3 significantly downregulated ribosomal dysfunction-enhanced tumor cell survival (Fig. 6E). In contrast, IGFBP3-suppressed cells displayed enhanced survival in response to 5-FU.*"

5. It is necessary to provide some insights into molecular mechanisms by which PRDM1 regulates the IGF/non-canonical Wnt pathway.

Response: As a reviewer pointed out, we performed additional mechanism studies **(Fig. 9C-F)** which were denoted as "Line 283: *Next, we addressed the signaling mechanism of PRDM1-associated differential regulation of chemoresistance. Ribosome-inactivated cells displayed increased levels of phospho-GSK3β (pGSK3β), an inactive form of GSK3β indicating active Wnt/β-catenin signaling (Fig. 9C). In addition, levels of RhoA protein and phospho-JNK (pJNK), indicating Wnt/PCP signaling activation, were also elevated by ribosomal dysfunction. Both RIS-activated canonical and non-canonical Wnt-linked signaling pathways were dependent on PRDM1. Furthermore. PRDM1-regulated transcriptional machinery was further investigated using the genome-wide microarray data of human CRC cells. In particular, caudal type homeobox transcription factor 2 (CDX2) was identified as a downstream target of PRDM1. CDX2 is known to be involved in differentiation of the intestinal stem cells into enterocytes 36 and its deficiency is closely linked to increased risk of CRC 37. PRDM1-deficient cancer cells displayed marginal expression of CDX2 in response to ribosomal dysfunction, indicating a positive regulation of CDX2 by PRDM1. In spite of suppressed PRDM1, overexpression of CDX2 restored levels of Wnt/PCP signaling molecules (RhoA and pJNK) while attenuating Wnt/β-catenin signaling activation (pGSK3β) (Fig. 9C). This differential regulation of Wnt*

signaling molecules during ribosome-inactivating stress was similar to that in clinical transcriptomic profile of CRC from the TCGA dataset (n = 440, COAD). High PRDM1 group displayed enhanced levels of Wnt5A, FZD4, RhoA and ROCK1, which are mainly involved in the Wnt/PCP signaling activation (Fig. 9D). In contrast, subjects with high PRDM1 had low expressions of Wnt/β-catenin positive regulators (MYC and AXIN2) and high levels of Wnt/β-catenin negative regulators (APC and Dickkopf3 (DKK3)) (Fig. 9E). Therefore, PRDM1 expression was positively associated with Wnt/PCP rather than canonical Wnt/β-catenin pathways in patents with CRC."

Comments of Reviewer 2

The article of Juil Kim and Yuseok Moon entitled "Ribosomal alert-induced PRDM1 spoils anticancer actions via stemness regulation" is an interesting and solid work. Before this article can be published in Communications Biology some corrections are needed.

My concerns are:

1. The authors have to introduce abbreviation when they are first used; e.g. line 35.

Response: As a reviewer pointed out, we checked all abbreviations for the first used and fixed them.

2. In line 131 the authors mention that they used 30 biopsies from CRC patients but only two cases are presented in Figure 2.

Response: As a reviewer pointed out, quantitative analysis of all 30 biopsies from CRC patients were depicted in the **figure 3A graph** and supplementary **figure S2** in a paired way.

3. In line 159 the text does not fit to the cited Figure. The authors must be much more accurate.

Response: As a reviewer pointed out, we double checked the mislabeling to be consistent with the figure numbering. Since all figures were split for clear illustration, all numbering was fixed.

4. Line 190: The mentioned Figure 3E right part must be presented as Table 1 and should be deleted from the Figure 3.

Response: As a reviewer pointed out, we transformed the table into figure format since it reflect the color density-based quantitative values as a figure data ***(Fig. 5E).***

5. Starting with line 432 the "Material and methods" part significantly decrease in quality. The authors have to increase the second part of this chapter significantly. Information about dilution of antibodies, or even the used antibodies (line 524) and the cities where the company is located are missing. Sometimes the authors don´t provide the company from which they have the reagents. The authors have to provide this information.

Response: As a reviewer pointed out, we checked the missing information of materials in detail.

6. What want the authors say with "m" in line 486? Normally "m" is an abbreviation for metres but that makes no sense at all here.

Response: As a reviewer pointed out, "m" is the abbreviation for minutes. All other abbreviations including "s" and "h" were also inspected again.

7. In lines 787-788 the authors have to be more specific.

Response: As a reviewer pointed out, we wrote more specific as "Line 822: *indicate PRDM1 protein and mRNA expression in injected cancer cells*".

8. The authors have to organise the figures in a better way – this is the worst part of the whole manuscript (that has a high quality). In general Figures are overloaded and not well organized/structured. Figure 1: Blots are too small; part F and G should be fused together and combined as one part. Figure 2: Blots are too small; abbreviation in part A must be explained. Figure 3: Part C is too small. The authors should think about the possibility to present their interesting findings in several better organized figures and not in such a crowded way. The only

acceptable figure is S2.

Response: As a reviewer pointed out, we totally agree with this comment. All figures were split into 2-3 parts to enhance readability and illustration (***Totally 9 figures***). In case of Figure 2, we separated two sets since sub-experiment A and B are independently performed with different material combination.

REVIEWERS' COMMENTS:

Reviewer #1 (Remarks to the Author):

The authors have satisfactorily addressed all my comments on the original submission. I recommend the manuscript for publication.
